# A Systematic Review of Transcriptional Dysregulation in Huntington’s Disease Studied by RNA Sequencing

**DOI:** 10.3389/fgene.2021.751033

**Published:** 2021-10-15

**Authors:** Bimala Malla, Xuanzong Guo, Gökçe Senger, Zoi Chasapopoulou, Ferah Yildirim

**Affiliations:** ^1^ Department of Psychiatry and Psychotherapy, Charité-Universitätsmedizin Berlin, Berlin, Germany; ^2^ Department of Experimental Oncology, IEO European Institute of Oncology IRCCS, Milan, Italy

**Keywords:** huntington’s disease, transcriptional dysregulation, RNA sequencing, transcription factors, epigenetic regulators

## Abstract

Huntington’s disease (HD) is a chronic neurodegenerative disorder caused by an expansion of polyglutamine repeats in exon 1 of the Huntingtin gene. Transcriptional dysregulation accompanied by epigenetic alterations is an early and central disease mechanism in HD yet, the exact mechanisms and regulators, and their associated gene expression programs remain incompletely understood. This systematic review investigates genome-wide transcriptional studies that were conducted using RNA sequencing (RNA-seq) technology in HD patients and models. The review protocol was registered at the Open Science Framework (OSF). The biomedical literature and gene expression databases, PubMed and NCBI BioProject, Array Express, European Nucleotide Archive (ENA), European Genome-Phenome Archive (EGA), respectively, were searched using the defined terms specified in the protocol following the PRISMA guidelines. We conducted a complete literature and database search to retrieve all RNA-seq-based gene expression studies in HD published until August 2020, retrieving 288 articles and 237 datasets from PubMed and the databases, respectively. A total of 27 studies meeting the eligibility criteria were included in this review. Collectively, comparative analysis of the datasets revealed frequent genes that are consistently dysregulated in HD. In postmortem brains from HD patients, *DNAJB1, HSPA1B* and *HSPB1* genes were commonly upregulated across all brain regions and cell types except for medium spiny neurons (MSNs) at symptomatic disease stage, and *HSPH1* and *SAT1* genes were altered in expression in all symptomatic brain datasets, indicating early and sustained changes in the expression of genes related to heat shock response as well as response to misfolded proteins. Specifically in indirect pathway medium spiny neurons (iMSNs), mitochondria related genes were among the top uniquely dysregulated genes. Interestingly, blood from HD patients showed commonly differentially expressed genes with a number of brain regions and cells, with the highest number of overlapping genes with MSNs and BA9 region at symptomatic stage. We also found the differential expression and predicted altered activity of a set of transcription factors and epigenetic regulators, including *BCL6, EGR1, FOSL2* and *CREBBP, HDAC1, KDM4C*, respectively, which may underlie the observed transcriptional changes in HD. Altogether, our work provides a complete overview of the transcriptional studies in HD, and by data synthesis, reveals a number of common and unique gene expression and regulatory changes across different cell and tissue types in HD. These changes could elucidate new insights into molecular mechanisms of differential vulnerability in HD.

**Systematic Review Registration:** https://osf.io/pm3wq

## Introduction

Huntington’s disease (HD) is a dominantly inherited neurodegenerative disorder caused by an expansion in CAG repeats in the exon I of the *Huntingtin* gene (*HTT*) (MacDonald, 1993). The mutant *HTT* gene, which translates into an abnormally long polyglutamine tract in the Huntingtin protein is expressed ubiquitously and leads to brain region-specific progressive neuronal dysfunction and degeneration that primarily affects the striatum during early disease stages and spreads to other brain regions as the disease progresses (Tabrizi et al., 2012). The disease is characterized by motor symptoms such as involuntary choreic movements, dystonia and rigidity, and is associated with psychiatric alterations, including apathy, irritability, depression and anxiety that often precede the onset of motor symptoms (Paulsen et al., 2005; Duff et al., 2007; Gil and Rego, 2008). Although many of the motor and psychiatric features of HD can be symptomatically treated (Mason and Barker, 2009; Anderson et al., 2018), a disease-modifying treatment to slow or stop the disease progression is still not available for HD patients.

While the genetic cause of HD has been known for almost 3 decades, the molecular mechanisms underlying the disease pathogenesis are complex, involving disruption of multiple cellular processes, among which transcriptional dysregulation is one of the earliest and central pathogenic mechanisms. Transcriptional profiling studies demonstrated progressive changes in gene expression in HD human brain and in experimental disease models (Hodges et al., 2006; Kuhn et al., 2007; Seredenina and Luthi-Carter, 2012; Vashishtha et al., 2013; Yildirim et al., 2019). Transcriptional dysregulation of many neuronal genes, including neurotransmitters, neurotrophins and their receptors, as well as those that are related to stress-response pathways and cell death were reported in HD brain. Some of the key neuronal genes that are consistently shown to be repressed across human HD brain and in animal models include brain-derived neurotrophic factor (*Bdnf*), dopamine receptor 2 (*Drd2*), dopamine receptor 1a (*Drd1a*), preproenkephalin (*Penk1*), adenosine A2a Receptor (*Adora2a*) and protein phosphatase 1 regulatory subunit 1B (*Ppp1r1b*).

Mechanisms through which mutant *HTT* has been proposed to cause transcriptional dysregulation include sequestration as well as soluble interactions with regulators of transcription leading to perturbation of their activities (Helmlinger et al., 2006; Cha, 2007; Ross and Tabrizi, 2011). In close relation to the changes in transcription and regulatory activities, epigenetic status is also altered in HD (Sadri-Vakili et al., 2007; Hervas-Corpion et al., 2018). In previous genome-wide studies, we found large changes in DNA methylation and key histone modifications such as H3K4 trimethylation, H3K27 acetylation and H3K36 trimethylation in cell and mouse models as well as in iPSC-derived neurons and postmortem brains from HD patients. These changes were linked to altered activities of certain transcription factors in HD (Ng et al., 2013; Vashishtha et al., 2013; Consortium, 2017). Importantly, genetic and pharmacological approaches that target epigenetic changes to correct aberrant transcription were shown to ameliorate HD features in preclinical trials (Ferrante et al., 2003; Thomas et al., 2008; Vashishtha et al., 2013; Suelves et al., 2017; Yildirim et al., 2019; Hecklau et al., 2021), validating the central role of transcriptional dysregulation in HD pathogenesis. Collectively, others and we demonstrated aberrant expression of thousands of genes and the associated changes in epigenomic profiles and regulatory factor activities in cell and animal models of HD and in HD patients; however, a systematic review comprising all the transcriptional profiling studies in HD is still lacking.

Here, we reviewed 27 independent studies on cellular models in human, human brain and blood as well as in non-human models. Within the human models, we observed large differences in gene expression, enrichment of gene sets and the related processes and pathways. While cellular models showed enrichment of cellular and developmental processes, postmortem brain models showed enrichment of genes involved in heat shock response, response to protein unfolding, synaptic transmission among others. One of the human studies that compared presymptomatic *versus* symptomatic brain provided valuable information regarding the striking uniqueness of differentially expressed genes (DEGs) between the presymptomatic and symptomatic brain. We observed that the heat shock response genes were significantly upregulated in presymptomatic brain. In particular, three common genes among all the postmortem brain regions, except for symptomatic MSNs, *DNAJB1*, *HSPA1B* and *HSPB1* were upregulated. However, this upregulation was less prominent in the symptomatic brain regions, and was not observed in the striatum in mouse studies. Within the striatum, MSNs exhibit dysregulation of mitochondrial function-related genes in addition to *SAT1* gene that is common with all the late postmortem brain categories. Additionally, blood shared more DEGs with symptomatic postmortem brain than with presymptomatic. Furthermore, non-human datasets also shared some key DEGs like, *Adora2a, Adcy5, CamkV, Penk* that are associated with HD.

## Methods

### Systematic Review Methodology

To retrieve all RNA-seq differential gene expression studies on HD patients or models, we searched PubMed and data repositories in a systematic way. The following term was used to search PubMed with the Advanced Search function ((huntingt*[MeSH Terms] OR huntingt*[tiab]) AND (Sequence Analysis, RNA [MeSH Terms] OR High-Throughput RNA Sequencing [MeSH Terms] OR Gene Regulatory Networks/genetics* [MeSH Terms] OR Genomics/methods* [MeSH Terms] OR RNA/genetics* [MeSH Terms] OR Gene Expression Profiling [MeSH Terms] OR Transcriptome* [MeSH Terms] OR Transcriptome/genetics* [MeSH Terms] OR RNA-seq*[Title/Abstract] OR “transcriptome profiling” [tiab] OR “transcriptome analysis” [tiab] OR “transcriptional dysregulation” [tiab] OR “genome-wide expression profiling” [tiab] OR “genome-wide expression analysis” [tiab] OR “differential* express*” [tiab] OR “mRNA-seq” [tiab] OR “transcriptome sequencing” [tiab] OR “transcriptional signatures” [tiab] OR “transcriptional alterations” [tiab] OR “transcriptional changes” [tiab]) NOT (review [pt])) AND ((“2013/01/01” [Date - Publication]: “2020/08/15” [Date-Publication])). For data repositories, search terms were adapted to each database: 1) huntingt*[title] AND “transcriptome gene expression” [Filter] for BioProject (SRA and GEO); 2) huntingt* AND exptype:“RNA-seq of coding RNA” for ArrayExpress; 3) huntingt* AND RNA-seq, with filter “study” for European Nucleotide Archive; 4) huntingt* for European Genome-Phenome Archive. In total, 288 articles were identified through PubMed and 237 datasets were identified through data repositories. Then, the articles and datasets were screened with the following exclusion criteria in order of priority: 1) Non-HD study, 2) Erratum/Superseries, 3) Review, 4) Different analysis of same data, 5) Non-RNA study, 6) RNA isoform/noncoding RNA study, 7) *HTT* knockout/-down study, 8) Treatment study, 9) Microarray, 10) Non-RNA-seq study, 11) Non-peer reviewed and 12) DEG not available. The screening process is presented in a PRISMA flow diagram (Supplementary Figure S1).

### Data Extraction

For human HD datasets for which all filtered expressed genes were not provided (*n* = 12), lowly expressed genes were removed from the expression matrix based on the selection criteria defined in each paper. For each subcategory, background gene lists were created by taking the union of all expressed genes in the datasets within the corresponding category.

### Human Samples

In total, 14 HD RNA-seq studies were performed on samples from either human cell culture models, blood samples or post-mortem brain samples. Cell culture models were derived from either embryonic stem cells or induced pluripotent stem cells, and samples were collected at the pluripotent, multipotent, or terminally differentiated stage. Two HD blood studies were included, one with monocytes from Grade 0–2 HD patients, one with platelets from all stages of HD. Post-mortem brain samples come from asymptomatic and symptomatic HD patients, specifically, caudate/putamen, motor cortex, prefrontal cortex and cingulate cortex. We analyzed 44 datasets after excluding 6 astrocyte datasets that didn’t analyze HD vs. WT differential expression. Then, based on sample origin, tissue type and disease stage, human HD datasets were grouped into 11 categories-cell culture datasets included pluripotent, progenitor, differentiated_neuronal, and differentiated_non_neuronal; post-mortem brain datasets included brain_early_striatal, brain_early_cortical, brain_late_striatal_neuronal_MSN, brain_late_striatal_neuronal_IN, brain_late_striatal_non_neuronal and brain_late_cortical; and the blood.

### Non-Human Samples

For non-human analyses, 14 HD RNA-seq studies from mouse, monkey and sheep models were included. HD mouse models included R6/1, R6/2, HdhQ150/175, BACHD, CHL2 and Swiss Webster mouse; mouse cell lines included E14 STHdhQ111 and BV2 microglia; other HD models included Rhesus-macaque pluripotent cell lines and OVT73 sheep. Then, we graded mouse HD datasets as early, intermediate and late stages based on prior literature. The datasets have been graded as follows. R6/1: 1 and 2 months-early, 7.5 months-late. R6/2: 1 month-early, 2–3.75 months-late. HdhQ150: 8 months-intermediate, 22 months-late. HdhQ175: 2 months-early, 6 months-intermediate, 10 months-late. CHL2: 12 months-intermediate. BACHD: 1 month-early.

### Gene Frequency and Overlap Analyses

The representative differentially expressed genes (DEGs) for all mouse and human categories were defined based on how often a gene was observed as significantly changed across the datasets in each category. Genes that are differentially expressed in at least two datasets were included in the category-representative DEGs. For subcategories with single dataset, all the genes were considered. As an exception, for the human blood category, union of DEGs from the two datasets within the blood category were considered as blood category-representative DEGs as the number of overlapping genes between the two blood datasets is 1.

Gene overlap analysis between pairwise comparisons were performed by using one-sided Fisher’s exact test. To test gene overlap among more than two categories, we used the SuperExactTest (Wang et al., 2015) package in R. Background gene list for each category was defined as the union of all expressed genes in each dataset. All analyses were performed in R.

### GSEA Analysis

Gene set enrichment analysis (GSEA) (Subramanian et al., 2005), for differentially expressed genes between HD and control samples, was performed for each HD human dataset (*n* = 44) with the GSEA method in the WebGestaltR package v.0.4.2 (Liao et al., 2019) for the following functional databases; KEGG pathway and Gene Ontology Biological Process. Differentially expressed genes were ranked based on their log fold changes with HD over wild type.

### GO Analysis

Common or unique gene sets were analyzed by online Gene Ontology enRIchment anaLysis and visuaLizAtion tool (GOrilla) (Eden et al., 2009). For pairwise comparisons, the intersection between the two datasets’ backgrounds was used as the background set. For multiple comparisons in the subcategories, the subcategory background was used as the background set. For unique genes in a subcategory or dataset, the subcategory or dataset background was used as the background set. Statistical significance was set at FDR <0.05.

### Regulator Analysis

To identify potential regulators for DEGs in each HD human dataset, we used hTFtarget database in which human TFs were curated from Chip-Seq experiments in different cell lines, tissues and cells (Zhang et al., 2020b). For each HD human dataset, DEGs were ranked based on absolute logFC and then top 500 DEGs were queried for potential regulators. For the datasets that have less than 500 DEGs, all DEGs were considered for the query. After obtaining potential regulators for each dataset, further filtering was performed, in which regulators that were not found in all expressed gene list of the corresponding dataset were removed. For the datasets that do not have all expressed gene lists, tissue specific gene lists were curated from the (Uhlen et al., 2015) and used for the filtering. An activity score was calculated for each regulator as a ratio of the number of DEGs a regulator controls to the total number of DEGs queried.

The overlap analysis between the TFs obtained from H3K27ac motif data from the HD iPSC Consortium 2017 dataset and the regulators obtained from hTFtarget database for the same study was performed by using Fisher’s exact test. All expressed genes from the same dataset were used as a background.

### Epigenetic Modifiers and Transcription Factors

Human TFs (*n* = 2,765) and epigenetic modifiers (*n* = 167) were curated from The Human Transcription Factors website (Lambert et al., 2018) and dbEM database (Singh Nanda et al., 2016), respectively. Among 2,765 curated proteins classified as TF with a known motif, TF with an inferred motif, likely TF, ssDNA/RNA binding protein, or unlikely TF, we only considered the following classes; TF with a known motif, TF with an inferred motif, which revealed 1,639 known and human TFs. Then, differential expression status of the curated human TFs and epigenetic modifiers were checked in all 44 HD human datasets.

## Results

**FIGURE 1:**
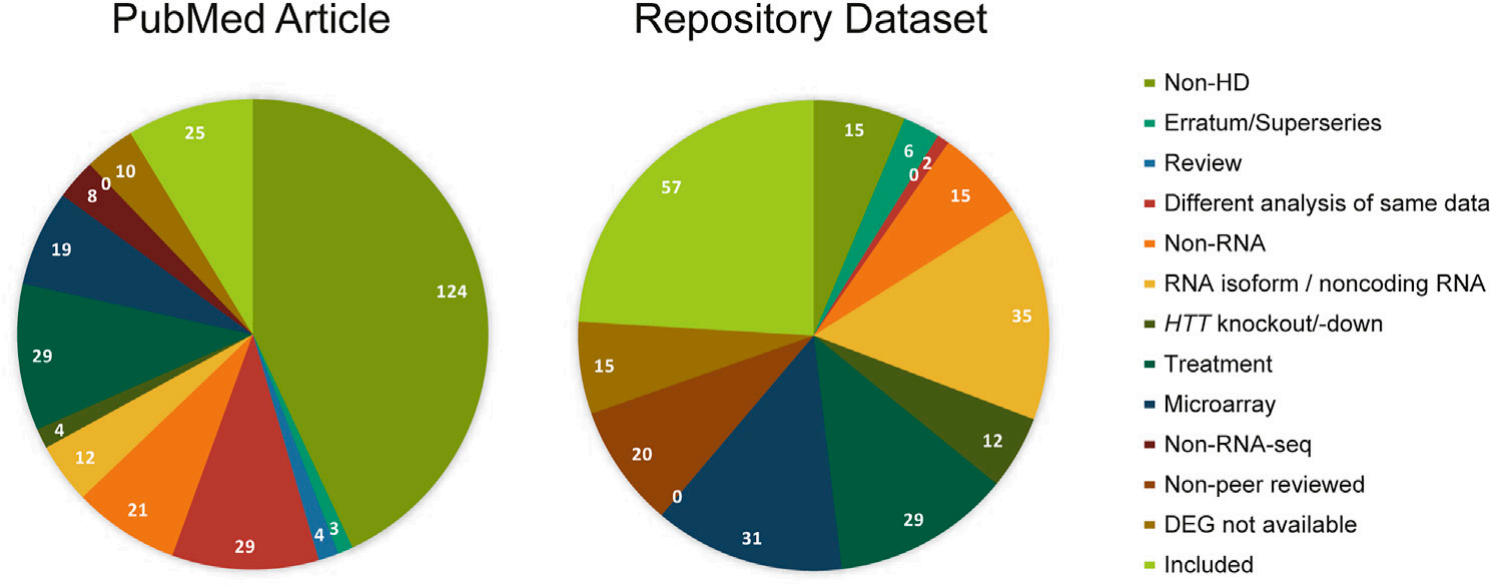
Studies screened from PubMed and dataset repositories. PubMed articles and repository datasets are screened with the same exclusion criteria in decreasing order of priority: non-HD, erratum (Pubmed)/superserie entries (repository dataset), review, different analysis of same data, non-RNA, RNA isoform/noncoding RNA, HTT knockout/down, treatment, microarray, non-RNA-seq, and no DEG analysis. The numbers of PubMed articles or repository datasets in each category are indicated.

In total, 288 PubMed articles and 237 datasets published until August 2020 were retrieved using our predefined search terms that focused on transcriptional profiling studies using RNA sequencing in Huntington’s disease. Of these, 25 PubMed articles and 57 datasets met our inclusion criteria, while others were excluded based on 11 preset exclusion criteria, as described in the methods section (Figure 1). Redundancy in datasets between the four data repositories, BioProject, ArrayExpress, European Nucleotide Archive (ENA) and European Genome-Phenome Archive (EGA), was identified resulting in 28 unique datasets out of 57. Then, 28 datasets were traced to their corresponding publications, and we found 20 unique publications. Among 25 PubMed and 20 dataset related publications, 18 publications were common. In the end, 27 publications fulfilled all the criteria and were included in this systematic review (Supplementary Figure S1; Supplementary Dataset S1).

### Transcriptional Changes in Human HD

This review included 44 differentially expressed gene (DEG) datasets from 14 human HD studies (Agus et al., 2019; Al-Dalahmah et al., 2020; Consortium, 2017; Denis et al., 2019; Lee et al., 2020; Lim et al., 2017; Lin et al., 2016; Mehta et al., 2018; Miller et al., 2016; Ooi et al., 2019; Osipovitch et al., 2019; Ring et al., 2015; Smith-Geater et al., 2020; Switonska et al., 2018) (Table 1). The hierarchical clustering of all 44 datasets showed clustering of datasets irrespective of the differences in the origin of tissue/sample (Supplementary Figure S2) (Ooi et al., 2019; Lee et al., 2020). Thus, we based our analyses on grouping of the datasets according to tissue/cell type and disease stage.

**TABLE 1:** Characteristics of human HD studies included in this review. The table summarizes PMID, Sample, Cell Type, Reference and Key Findings of each included study (Ring et al., 2015; Lin et al., 2016; Miller et al., 2016; Consortium, 2017; Lim et al., 2017; Mehta et al., 2018; Switonska et al., 2018; Agus et al., 2019; Denis et al., 2019; Ooi et al., 2019; Osipovitch et al., 2019; Al-Dalahmah et al., 2020; Lee et al., 2020; Smith-Geater et al., 2020).

PMID	Sample	Cell type	References	Key findings
30554964	hESC	Glial/Astrocytic progenitor cell	Osipovitch et al. (2019)	Found downregulation in glial/astrocyte differentiation and myelin synthesis
30811996	hESC	PSC, neural progenitor cell, neuron, hepatocyte and myocyte	Ooi et al. (2019)	Found CAG repeat length-related abnormalities in mitochondrial respiration and oxidative stress and enhanced susceptibility to DNA damage; also found cell-type-specific molecular phenotypes
32109367	hiPSC	Medium spiny neuron	Smith-Geater et al. (2020)	Found a persistent cyclin D1+ neural stem cell (NSC) population selectively in adult-onset HD iPSCs during differentiation, which can be rescued by WNT inhibitor
28514657	hiPSC	Brain microvascular endothelial	Lim et al. (2017)	Found abnormality in angiogenesis and blood brain barrier properties
30713489	hiPSC	Pluripotent stem cell	Switonska et al. (2018)	Found mostly downregulation in DNA damage reponse and apoptosis, potentially linked to TP50; found upregulation in embryogenesis and early neural development
30355486	hiPSC	Cortical neuron	Mehta et al. (2018)	Found altered transcriptomics, morphology and electrophysiological maturation
28319609	hiPSC	Neural	Consortium, (2017)	Found downregulation in glutamate and GABA signaling, axonal guidance and calcium influx, which can be rescued by isoxazole-9
26651603	hiPSC	Pluripotent/Neural stem cell	Ring et al. (2015)	Found dysregulation in TGF-b and netrin-1 pathway
27170315	Blood	Primary monocyte	Miller et al. (2016)	Found upregulation in proinflammatory cytokines such as IL6, potentially resulting from activation of NFkB pathway
30567722	Blood	Platelet	Denis et al. (2019)	Found dysregulation in angiogenic factor release, thrombosis, angiogenesis and vascular haemostasis
31619230	Brain	Caudate; BA9 prefrontal cortex	Agus et al. (2019)	Found transcriptomic concordance between prodromal HD caudate nucleus and symptomatic HD BA9; found dysregulation in heat shock response, particularly HSPA6 and HSPA1A, with NPAS4 and REST1/2 being potential early responders
27378699	Brain	BA4 motor cortex	Lin et al. (2016)	Found 593 differential splicing events and 4 differentially expressed splicing factors including PTBP1
32681824	Brain	Caudate/Putamen	Lee et al. (2020)	Found release of mitochondrial RNA and upregualtion in innate immune signaling
32070434	Brain	Cingulate cortex	Al-Dalahmah et al. (2020)	Found downregulation of protoplasmic astrocyte function and lipid synthesis in astrocytes, which could be divided in 3 reactive states

**FIGURE 2:**
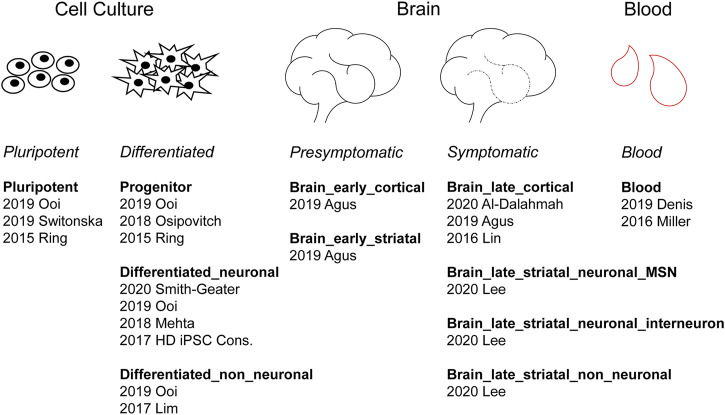
Categorization of human HD differentially expressed gene (DEG) datasets. According to the sample source, human HD studies can be divided into three groups: cell culture, brain and blood. Cell culture studies can be further divided into pluripotent and differentiated subgroups. Brain studies can be divided into presymptomatic and symptomatic subgroups. Under each subheading are listed the respective studies identified by their publication year and the last name of the first author.

**FIGURE 3:**
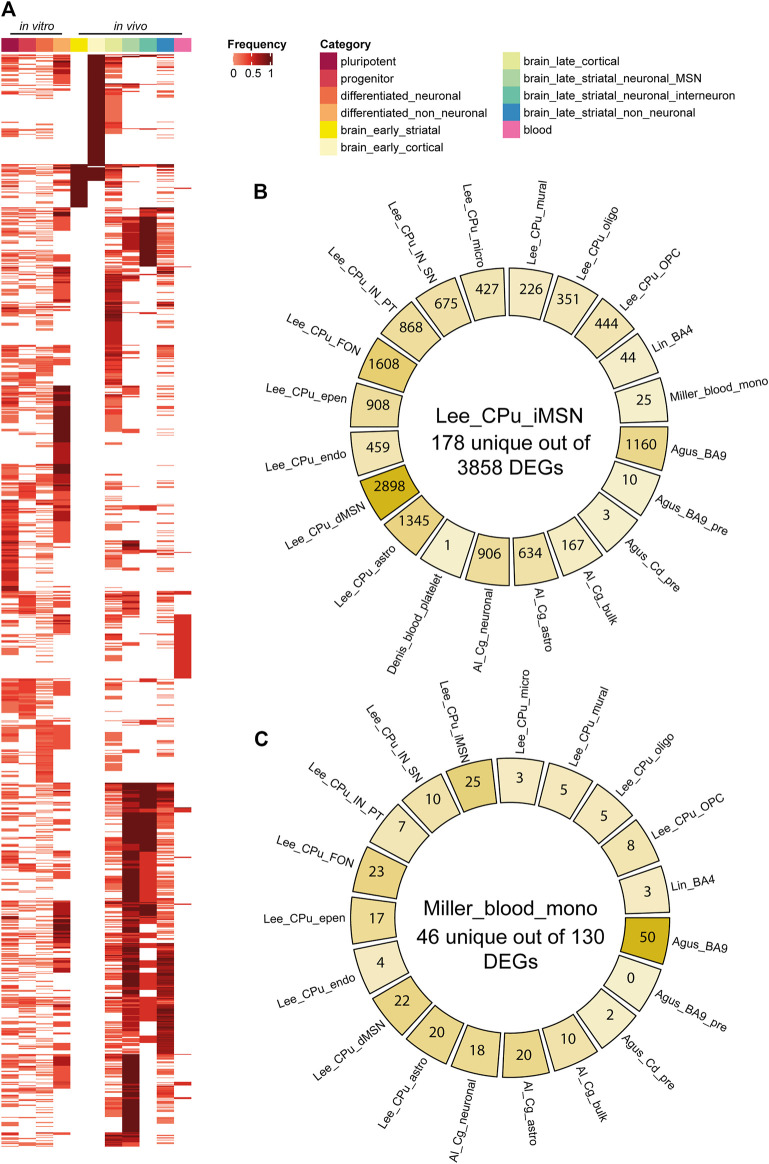
HD differentially expressed gene (DEG) comparisons between categories and datasets. **(A)** DEG Frequency heatmap across all subcategories. Each column is a subcategory, each row is a DEG. DEG frequency is calculated as the number of datasets that DEG appears in divided by the total number of datasets in that subcategory. **(B,C)** Diagrams for pairwise comparisons of indirect pathway medium spiny neuron (iMSN) or blood monocyte DEG list vs all other data from primary tissues/cells. At the center is the number of unique DEGs in iMSN or blood monocyte, and on the side is the number of DEGs in common with other datasets. Abbreviations: CPu, caudate putamen; astro, astrocyte; epen, ependymal; endo, endothelial; IN, interneuron; PT, Pvalb/Th-expressing; MSN, medium spiny neuron; MNS, MAP2+ and NES/SOX2-; OPC, oligodendrocyte progenitor cell; oligo, oligodendrocyte; micro, microglia; NSC, neural stem cell; Cg, cingulate cortex; FON, Foxp2/Olfm3-expressing neuron; SN, Sst/Npy-expressing; dSPN/iSPN, direct/indirect pathway spiny projection neuron; mono, monocyte; NPC, neural progenitor cell; CoNeuron, cortical neuron; Cd, caudate nucleus; pre, presymptomatic; GPC, glial progenitor cell; APC, astrocyte precursor cell; BBB, blood brain barrier.

We classified 44 human DEG datasets into five categories based on sample source and disease stage (Figure 2). The five groups were further categorized into the following 11 subcategories: pluripotent, progenitor, differentiated neuronal and differentiated non-neuronal cells from cellular models; brain_early_striatal, brain_early_cortical, brain_late_cortical, brain_late_striatal_neuronal_MSN, brain_late_striatal_neuronal_interneuron and brain_late_striatal_non-neuronal from brain tissue and blood. The top upregulated and downregulated genes in each subcategory are shown in Table 2. There were no commonly differentially expressed genes (DEGs) across all the studies. We then identified frequently dysregulated genes in each subcategory by taking the genes that appeared in at least two of the datasets. Figure 3A shows a heatmap of the frequent DEGs across all the subcategories and shows that DEGs from early cortical tissues changed extensively in the late cortical tissues from symptomatic brains (Figure 3A, Supplementary Figure S3). The late striatal subcategories showed similar differential expression of many genes. Additionally, we observed some similarities among pluripotent, progenitor, differentiated_neuronal and differentiated_non_neuronal cellular subcategories.

**TABLE 2:** Human top differentially expressed genes (DEGs) in each category. The table summarizes the top DEGs in brain subcategories in human, first ranked by the frequency of DEGs, then by the average FDR value of DEGs across all datasets in that subcategory.

Category	Top 20 DEGs
Pluripotent	XDH, TRIM69, ALG10B, POU6F2, AC005276, SLC24A3, PI15, CNTNAP3B, RFTN2, FLRT2, PLCB1, C3, RP11-78L16, SAMD15, KCNMA1, CBSL, KC6, TRPV4, TMEM132C, VLDLR
Progenitor	HSPA2, SERPINE2, SERPINI1, MDGA2, FRZB, SIRT2, SRRM4, FAM64A, YWHAE, LRRC4B, INSIG1, FGFBP3, ZNF718, LINGO1, COMMD7, SNX10, NEFM, DPP6, DMGDH, NFASC
Differentiated_neuronal	CDKN1A, VGLL3, ALG10B, INPP5D, TP53, FST, LINC01021, COL14A1, XIST, MT1E, FGF14, RP11-706O15, PTGER3, FAS, SCN9A, NPIPA5, RP11-93G5, ZNF558, LHX8, PHLDA3
Differentiated_non_neuronal	CHCHD2, CYYR1, PCYT1B, SMN2, GNG4, PTHLH, SH3BP5, EFCAB2, TMEM51, ARHGAP8, IFITM2, CITED4, PMEPA1, RND3, FAM110A, MEX3C, HOXA3, C2CD2, TCF4, ATP13A2
Brain_early_cortical	FOSB, NPAS4, HSPA6, DNAJB1, JUN, HIST2H2AA4, PPP1R15A, SLC16A3, HSPA1A, SLC11A1, ADGRE2, AZGP1, CCL4, PLIN2, RNF122, LMNA, THEMIS2, SERPINE1, SPP1, FPR1
Brain_late_cortical	MT1M, MT1F, MT1G, MT1E, FKBP5, GFAP, FAM107A, CRYM, SLC14A1, CEBPD, RHOBTB3, SLC39A10, NEUROD6, TAC1, HSPB1, ID3, BAG3, MT2A, IL17RB, NUPR1
Brain_early_striatal	HSPA6, NPAS4, STC1, FP565260.3, DNAJB1, HSPA1A, BAG3, HSPA1B, NAPSB, NPIPB15, SERPINH1, SIK1, DEPP1, HSPB1, CCL19, ADM, RGS1, LTF, ATF3, ANGPT2
Brain_late_striatal_neuronal_MSN	NGEF, CCDC88C, PDE10A, RIC8B, SLC25A37, RYR3, STRIP2, EPB41L2, FNBP1L, LPP, RTTN, NETO1, SPON1, STXBP5L, JPH4, OTOF, PDIA6, KCNH4, ONECUT2, HRH2
Brain_late_striatal_neuronal_interneuron	SLC26A3, INO80D, RASGEF1B, GPHN, NEGR1, PRPF4B, ATP1B1, EML5, ALK, PCDH15, CDH10, NTM, TENM1, WSB1, PCP4, TENT5A, ST6GALNAC5, NRDC, LINGO1, PLCH1
Brain_late_striatal_non_neuronal	INO80D, SLC26A3, DNAJB1, PCDH9, CSMD1, RASGEF1B, HSP90AA1, LINGO1, PTGES3, HSPB1, EIF1, DDX5, FTH1, ACTG1, PLCG2, DPP10, H3F3B, TMSB4X, RPL15, LPP

#### Cellular Models

Of 8594, 3119, 5633 and 10120 total DEGs in each subcategory, we obtained 1,638, 174, 804 and 2,125 frequently dysregulated genes in pluripotent, progenitor, differentiated_neuronal and differentiated_non-neuronal subcategories, respectively (Supplementary Datasets S2, S3). Comparison of the gene frequency across four subcategories of the cellular models showed that two genes, Immunoglobulin Superfamily DCC Subclass Member 3 (*IGDCC3*) and XK Related 4 (*XKR4*) were common in all four subcategories (*p* = 2.3E-4) (Supplementary Dataset S4). Overall, cellular models showed an enrichment of gene sets related to phosphatidylserine exposure on apoptotic cell surface (Supplementary Dataset S5).

Among pluripotent, differentiated_neuronal and differentiated_non-neuronal categories, 39 genes (*p* = 5.7E-28) overlapped, with the most significant enrichment of GO terms related to low-density lipoprotein transport. We observed an overlap of 156 genes (*p* = 1.7E-49) between pluripotent and differentiated_neuronal while there were 285 common genes (*p* = 5.3E-48) between pluripotent and differentiated_non-neuronal. The overlapping genes between pluripotent and progenitor (43 genes, *p* = 4.6E-18) showed enrichment of GO terms such as regulation of cell differentiation, regulation of developmental process, regulation of nervous system development, and regulation of cellular component organization. There were 115 overlapping genes (*p* = 5.8E-15) between differentiated_neuronal and differentiated_non-neuronal while, we obtained 551 unique genes in differentiated_neuronal subcategory (Supplementary Dataset S3).

#### Postmortem Brain Tissues From HD Patients

We classified the postmortem human HD brain category into six subcategories as mentioned above. In early_ and late_cortical, early_ and late_ striatal_medium spiny neuron (MSN), late_ striatal_interneuron and late_ striatal_non-neuronal brain tissues, there were 226, 2,806, 70, 3414, 1988 and 1914 frequently differentially expressed genes, respectively (Supplementary Dataset S3).

Interestingly, Agus et al. (2019) examined presymptomatic and symptomatic brain tissue from HD patients (Agus et al., 2019). In presymptomatic tissues, in early striatal and cortical subcategories, we obtained 25 common genes (*p* = 1.2E-35), such as *HSPA6, DNAJB1, HSPA1B, HSPA1A, HSPB1, JUN* (Supplementary Dataset S4) showing enrichment of GO terms related to the response to unfolded protein, negative regulation of transcription from RNA polymerase II promoter in response to stress, chaperone-mediated protein folding, “*de novo*” posttranslational protein folding, negative regulation of inclusion body assembly and, regulation of apoptotic process (Supplementary Dataset S5). In the cortex, we observed an overlap of 59 genes (*p* = 1.87E-13) between early and late cortical tissues (Supplementary Dataset S4). Some of the common genes were *HSPA6, HSPA7, SERPINH1, HSPA1A, DNAJB1, SLC11A1, HSPA1B, HSPB1, SERPINA1, JUNB, EGR1, SNAI1, PTPN6, SLC2A5, SAT1* and *HSPE1* that are related to GO terms such as response to unfolded protein, chaperone cofactor-dependent protein refolding, regulation of apoptotic process and immune effector process.

Within the three subcategories of the late striatal group from Lee et al., 2020, there were 488 common genes (*p* = 2.1E-275), showing enrichment of various GO terms such as regulation of neuron projection development, regulation of neurogenesis, regulation of neuron differentiation, regulation of nervous system development, regulation of synapse organization, biological adhesion, axon guidance, vesicle localization and cyclic nucleotide catabolic process (Supplementary Dataset S5). Meanwhile, 1,287 common genes (*p* = 0) between MSNs and interneurons in striatum showed enrichment of GO terms such as modulation of chemical synaptic transmission, regulation of neuron projection development, regulation of synaptic plasticity, regulation of neurotransmitter levels, glutamate receptor signaling pathway, axon guidance, regulation of synaptic vesicle exocytosis, regulation of NMDA receptor activity, regulation of cation channel activity, cytosolic calcium ion transport, lysosomal transport, glutamate secretion, receptor localization to synapse, metal ion homeostasis, and regulation of GTPase activity. On the other hand, 2,127 unique genes in MSNs compared to interneurons show enrichment of cytoskeleton organization, cytoskeleton-dependent intracellular transport, cell projection organization, movement of cell or subcellular component, microtubule-based process as well as transport, plasma membrane bounded cell projection organization, positive regulation of GTPase activity, macromolecule modification and nervous system process to name some (Supplementary Dataset S5). While there were 84 common genes between MSNs and non_neuronal tissues (*p* = 1.4E-152), interneurons and non-neuron cells within striatum showed an overlap of 694 genes (*p* = 2.2E-143).

As medium spiny projection neurons of the indirect pathway (iMSN/iSPN) are the most vulnerable cell types to degeneration in HD patients (Reiner et al., 1988), we examined in detail the upregulated and downregulated genes in iMSNs in the dataset Lee_CPu_iMSN (Lee et al., 2020). Most of the top upregulated genes, *MT-CO2, MT-ND4L, MT-ND4, MT-ATP6, MT-CO3, SLC26A3, INO80D,* and *MT-ND1,* were related to mitochondrial complex I, cytochrome c oxidase and ATP synthase, while the top downregulated genes were *OTOF, TAC1*, *PILRB, JPH4, DDX24, MPHOSPH8* and *KNOP1* (Table 2, Supplementary Table S2) that are related to immune activation, kinases, RNA preprocessing and cell division. Then, we compared Lee_CPu_iMSN dataset (Lee et al., 2020) with other brain and blood datasets (Figure 3B). Among 3858 differentially expressed iMSN genes, 178 were unique. We observed highest commonality of iMSNs with the direct pathway medium spiny projection neurons (dMSN) with 2,898 common genes. Interestingly, only 10 genes- *AL117339, SLC16A7, SAT1, SLC39A10, HOMER1, HSPA1b, JUND, SPP1, UBC* and *SLC2A13-*and 3 genes-*CRYAB, HSPA1B* and *HSPH1*- were common with presymptomatic BA9 cortex and caudate brain regions, respectively. Moreover, 25 genes were common with blood monocytes (Figure 3B, Supplementary Dataset S6; Table 3).

**TABLE 3:** Overlapping differentially expressed genes (DEGs) with blood monocyte DEG list in each brain subcategory. The table shows common DEGs between blood monocytes and the six brain subcategories. No common DEGs between early_cortex dataset and blood monocytes.

Brain subcategory	Overlapping DEGs with blood monocyte
Early_striatal	CCL19, PTGS2
Late_striatal_MSN	ANXA11, FGFR1OP2, GK5, GTF2E2, HECW2, KTN1, LOXL2, NAT8L, NEK11, NME7, OTOF, PHTF1, PID1, PLCB4, PLXNB1, PPM1H, SGK1, SLC25A37, SOX5, TEFM, WASF1, ZDHHC2, ZNF654
Late_striatal_interneuron	HECW2, KTN1, NEK11, OTOF, PID1, PLCB4, PLXNB1, SERPINB9, SLC25A37, STAC, TEFM, WASF1, ZDHHC2, ZNF654
Late_striatal_non_neuronal	ANXA11, CHORDC1, EVC2, KANSL1L, NAMPT, NT5E, PLCB4, SDCCAG8, SGK1, SLC25A37, SNX25, SOX5, SPARC, TEFM, TTLL7, VCAN, VEGFA
Early_cortex	
Late_cortex	ATXN7L3, CDK2, CEP152, CHORDC1, DLL4, EDN1, EVC2, FAM111A, FGFR1OP2, FZD7, KCNJ15, KTN1, MAPRE3, NAT8L, PID1, PPBP, S100A12, SDCCAG8, SERPINB9, SGK1, SLC45A1, SMO, SPARC, STX1A, TTLL7, VCAN, VEGFA

Importantly, in the five brain subcategories except for the brain_late_striatal_neuronal_MSNs, three genes, DnaI Heat Shock Protein Family (Hsp40) Member B1 (*DNAJB1*), Heat Shock Protein Family A (Hsp70) Member 1B (*HSPA1B*) and Heat Shock Protein Family B (Small) Member 1 (*HSPB1*) were common. These genes encode for HSP40, HSP70 and HSPB1 proteins, respectively, showing an enrichment of GO terms like chaperone-mediated protein folding, response to unfolded protein and negative regulation of inclusion body assembly. The expression of *DNAJB1, HSPA1B* and *HSPB1* was upregulated in early BA9 and caudate nucleus. In late brain subcategories related datasets, the expression was less upregulated in comparison to the presymptomatic datasets (Table 4). Similarly, Heat Shock Protein Family H (Hsp110) Member 1 (*HSPH1*) and Spermidine/Spermine N1-Acetyltransferase 1 (*SAT1*) were common for all late-brain categories (Supplementary Dataset S4).

**TABLE 4:** Log fold change (LFC) of differentially expressed gene (DEG), DNAJB1, HSPA1B and HSPB1 in human and mouse primary tissue datasets. The table summarized Log2 fold changes of the three most commonly dysregulated genes in postmortem brain across human and mouse primary tissue datasets. Note that the differentially expressed genes are bolded (Vashishtha et al., 2013; Mielcarek et al., 2014; Achour et al., 2015; Ring et al., 2015; Langfelder et al., 2016; Lin et al., 2016; Miller et al., 2016; Consortium, 2017; Lim et al., 2017; Mehta et al., 2018; Pan et al., 2018; Switonska et al., 2018; Agus et al., 2019; Denis et al., 2019; Ooi et al., 2019; Osipovitch et al., 2019; Yildirim et al., 2019; Al-Dalahmah et al., 2020; Lee et al., 2020; Smith-Geater et al., 2020; Wertz et al., 2020).

		DNAJB1	HSPA1B	HSPB1			Dnajb1	Hspa1b	Hspb1
Human	Agus_CN_pre	**3.368501**	**3.31007**	**2.556777**	Mouse	Vashishtha_R6-2_Co_12w		**−1.87404**	
	Agus_BA9	**1.046449**	**1.38684**	**1.805144**		Mielcarek_R6-2_heart_15w	**−1.9086**		
	Agus_BA9_pre	**3.159802**	**2.856196**	**2.68096**		Langfelder_Hdh_Q175_Co_10m		**−0.605**	
	Lin_BA4	**0.633903**	**0.680928**	**0.983342**		Langfelder_Hdh_Q175_skin_6m	**0.239018**	**0.367294**	
	Lee_CN_dSPN	**0.130567**	0.096195	**0.131141**		Langfelder_Hdh_Q175_CPu_10m		**−0.542**	
	Lee_CN_iSPN	0.099918	**0.135557**	0.087858		Achour_R6-1_Cpu-Cb_30w	**0.322727**		
	Lee_CN_FON	0.069187	0.064238	0.082339		Lee_Hdh_Q175_TRAP_iSPN_6m	**0.886169**		
	Lee_CN_astro	**0.53597**	**0.155755**	0.850693		Lee_R6-2_TRAP_dSPN_9w	**0.594363**		
	Lee_CN_micro	**0.307794**	**−0.25759**	**0.23768**		Lee_R6-2_TRAP_iSPN_9w	**0.490803**		
	Lee_CN_OPC	**0.140988**	**0.134697**	**0.117899**		Pan_SWR-J_Q72_CoNeuron_5d	**0.641914**	**0.570635**	
	Lee_CN_oligo	**0.151425**	0.048845	**0.106426**		Wertz_R6-2_Cpu_11w	**0.395566**		
	Lee_CN_IN_SN	0.019236	0.139542	0.015702		Yildirim_CHL2_CPu_1y		**0.838849**	
	Lee_CN_IN_PT	**0.16843**	**0.105475**	**0.223659**					
	Lee_CN_endo	**0.917992**	0.165135	**0.842291**					
	Lee_CN_mural	**0.421188**	**0.369823**	**0.591541**					
	Lee_CN_epen	**0.79928**	**0.404143**	**1.041503**					
	Al_CC_astro	**1.175889**	**1.759981**	**2.263495**					
	Al_CC_neuronal	**1.340038**	**1.089038**	**1.988857**					
	Al_CC	1.238239	**1.55208**	**2.135469**					
	Miller_blood	0.063423	0.041156	−0.02496					
	Denis_blood	−0.38268	0.76156	−0.10778					

#### Peripheral Tissues From HD Patients

To investigate concordant changes between blood and brain, we compared 130 DEGs from the primary monocyte dataset from Grade 0–2 HD patients (Miller et al., 2016) with all the brain datasets. In Figure 3C, we show the overlap of differentially expressed genes in blood with the datasets in postmortem brain tissues. We obtained 46 unique genes, such as *IL12B, IL23A, CECR5, CCL8, CD300E, TSPAN33, CA13, MIR1249, CLDN1* and *HPSE* to name some that were differentially expressed only in blood monocytes (Figure 3C). Of the top 10 differentially expressed genes in blood, 4 upregulated genes, *FAM124A, IL19*, *C6orf65* and *IL23A* and 4 downregulated genes, *ZNF414, R3HCC1, PGAP3* and *FAM213B* were unique for blood (Supplementary Dataset S2). Early striatum dataset-Agus_Cd_pre had two overlapping genes with the blood dataset: C-C Motif Chemokine Ligand 19 (*CCL19*) and Prostaglandin-Endoperoxide Synthase 2 (*PTGS2*) (*p* = 0.07). Moreover, we observed significant overlap of 27 frequently dysregulated genes (*p* = 0.004) with late cortical subcategory (Table 3).

### Gene Sets and Pathways Dysregulated in Human HD

Next, we performed Gene Set Enrichment Analysis (GSEA) (Subramanian et al., 2005) on 44 human DEG datasets using the ranked lists of DEGs based on their log fold changes, Gene ontology biological processes and KEGG pathways databases using WebGestalt (Liao et al., 2019).

GSEA analysis showed strong negative enrichment of genes associated with p53 signaling pathway and cancer in pluripotent cells in Switonska_hiPSC_PSC_109Q (Switonska et al., 2018). Contrastingly, Ring_hiPSC_PSC did not show any enrichment of p53 signaling pathways and cancer in pluripotent cells. It showed exclusively negative enrichment of gene sets related to metal ion homeostasis, immune response, regulation of transcription regulatory region DNA binding, JAK-STAT cascade and peptidyl-tyrosine phosphorylation (Ring et al., 2015). Regarding the progenitor subcategory, the neural stem cell dataset from Smith_hiPSC_MSN_NSC had the largest number of enriched gene gets, with positive enrichment in gene sets such as ribosome, spliceosome, DNA replication, base excision repair, RNA transport, mismatch repair, p53 signaling pathway, and cell cycle, and with negative enrichment in gene sets such as synaptic vesicle cycle, oxidative phosphorylation, Alzheimer disease, and Parkinson disease (Smith-Geater et al., 2020). Smith_hiPSC_MSN_adult showed strong positive enrichment of numerous gene sets such as metal ion homeostasis, lysosome, JAK-STAT cascade, regulation of transcription regulatory region, cAMP signaling pathway, nerve development, synaptic transmission, Interestingly, Mehta_hiPSC_CoNeuron datasets showed strong negative enrichment of cancer related genes on day-0 in differentiated neuronal cell culture that changed on day-80 and 100 showing positive enrichment and no enrichment on day-130 (Mehta et al., 2018) (Supplementary Dataset S7). Most differentiated_neuronal datasets showed positive enrichment of metal ion homeostasis and detoxification.

**FIGURE 4:**
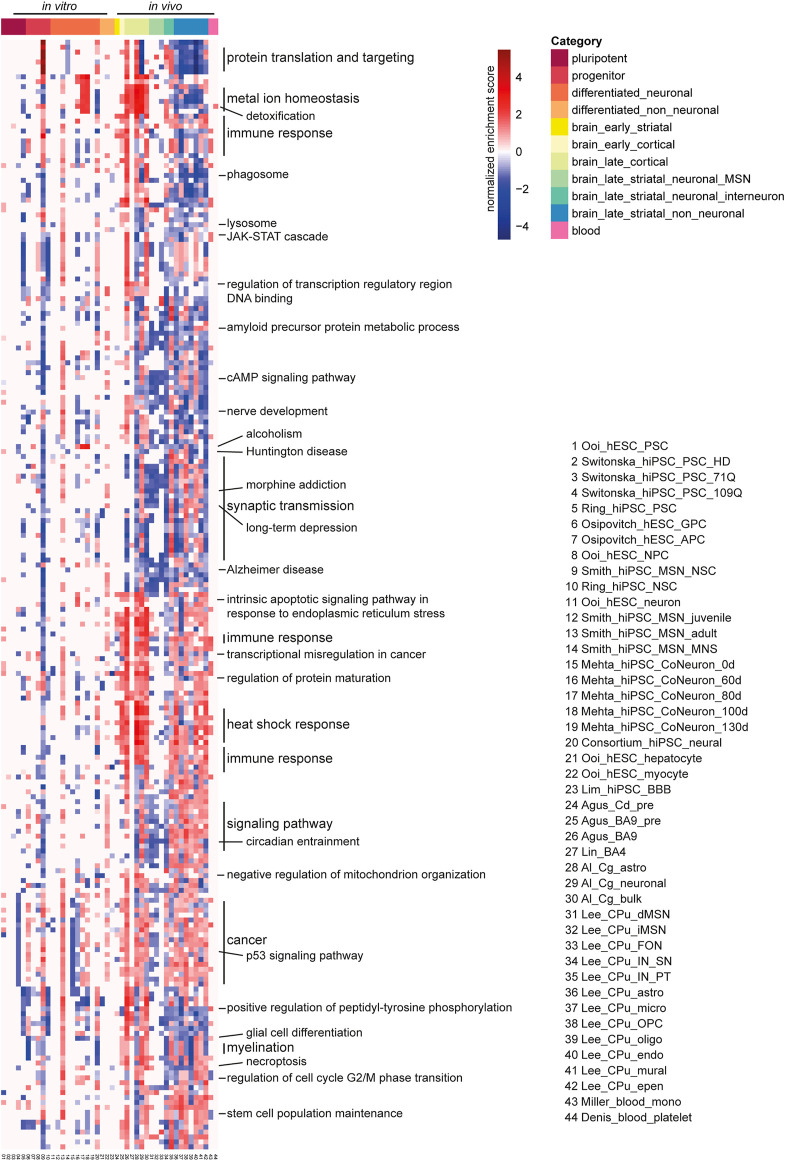
Gene set enrichment analysis (GSEA) enrichment heatmap of 44 human HD RNA-seq datasets. Top 20 enriched gene sets for the GO biological process and KEGG pathway were combined and shown here. Each column is a human HD RNA-seq DEG dataset, each row is a gene set of the GO biological process or KEGG pathway. Adjacent gene sets with a common biological function are grouped and labeled as such. Red indicates positive enrichment, blue negative. Abbreviations: CPu, caudate putamen; astro, astrocyte; epen, ependymal; endo, endothelial; IN, interneuron; PT, Pvalb/Th-expressing; MSN, medium spiny neuron; MNS, MAP2+ and NES/SOX2-; OPC, oligodendrocyte progenitor cell; oligo, oligodendrocyte; micro, microglia; NSC, neural stem cell; Cg, cingulate cortex; FON, Foxp2/Olfm3-expressing neuron; SN, Sst/Npy-expressing; dSPN/iSPN, direct/indirect pathway spiny projection neuron; mono, monocyte; NPC, neural progenitor cell; CoNeuron, cortical neuron; Cd, caudate nucleus; pre, presymptomatic; GPC, glial progenitor cell; APC, astrocyte precursor cell; BBB, blood brain barrier.

In brain, presymptomatic striatum and cortex showed positive regulation of gene sets associated with intrinsic apoptotic signaling pathway in response to endoplasmic reticulum stress, immune response, transcriptional misregulation in cancer, regulation of protein maturation, heat shock response. In symptomatic brain subcategory, the late cortex was positively enriched for various gene sets related to metal ion homeostasis, protein folding, p53 signaling, cancer and positive regulation of peptidyl-tyrosine phosphorylation. Late cortex and late_striatal_neuronal_MSNs showed common positive enrichment of glutathione metabolism, RNA polymerase, biosynthesis of unsaturated fatty acids, alanine, aspartate and glutamate metabolism, protein localization to chromosome/centromeric region, DNA replication, acidic amino acid transport, postsynapse assembly, purine metabolism, and synaptic vesicle cycle, while sharing negative enrichment in gene sets related to amyloid precursor protein metabolic process, cAMP signaling pathway, nerve development, alcoholism, Huntington disease, synaptic transmission, morphine addiction, long-term depression, Alzheimer disease and circadian entrainment (Figure 4, Supplementary Figure S5; Supplementary Dataset S7). In striatal interneurons, Sst/Npy-expressing (SN) GABAergic interneurons in Lee_CPu_IN_SN dataset and the Pvalb/Th-expressing (PT) GABAergic interneurons in Lee_CPu_IN_PT dataset revealed a contrasting enrichment profile of various gene sets (Figure 4, Supplementary Figure S5; Supplementary Dataset S7).

For the blood monocyte dataset- Miller_blood_mono, there was positive enrichment in gene sets such as cytokine-cytokine receptor interaction, chemokine signaling pathway, *IL-17* signaling pathway, *JAK-STAT* signaling pathway and pathways in cancer (Figure 4, Supplementary Figure S5). Concordant enrichment in gene sets, such as, chemokine signaling pathway and *IL-17* signaling pathway was found in early cortical dataset from Agus et al., 2019, as well as in most of the late cortical datasets and late striatal non-neuronal datasets from Lee et al., 2020. Positive enrichment in the gene set *JAK-STAT* signaling pathway was also found in the neural stem cell and adult-onset HD datasets from Smith-Geater et al., 2020, and most of the late cortical datasets and late striatal non-neuronal datasets (Smith-Geater et al., 2020). For the blood platelet dataset, the only enriched gene set was related to detoxification in the positive direction, and the same positive enrichment was found in neural stem cell and adult-onset HD datasets from Smith-Geater et al., 2020, the Day 60, Day 80 and Day 100 iPSC-derived cortical neuron datasets from Mehta et al., 2018, all the late cortical datasets and both of the interneuron datasets. Meanwhile, negative enrichment in the gene set detoxification was observed in the hiPSC pluripotent stem cell dataset from Ring et al., 2015, the neural dataset from HD iPSC Consortium 2017 as well as in the astrocyte, the microglia, the OPC, the oligodendrocyte, the endothelial cell and the ependymal cell datasets from Lee et al., 2020 (Ring et al., 2015; Consortium, 2017; Mehta et al., 2018; Lee et al., 2020; Smith-Geater et al., 2020) (Supplementary Dataset S7).

### Regulators of Transcription in Human HD

To investigate potential regulators responsible for differential expression of genes in HD, we searched for the potential regulators of the DEGs in each dataset using human transcription factors target (hTFtarget) database (Zhang et al., 2020a). An activity score based on the number of DEGs co-regulated by a regulator in a dataset were assigned for each regulator in the respective dataset.

**FIGURE 5:**
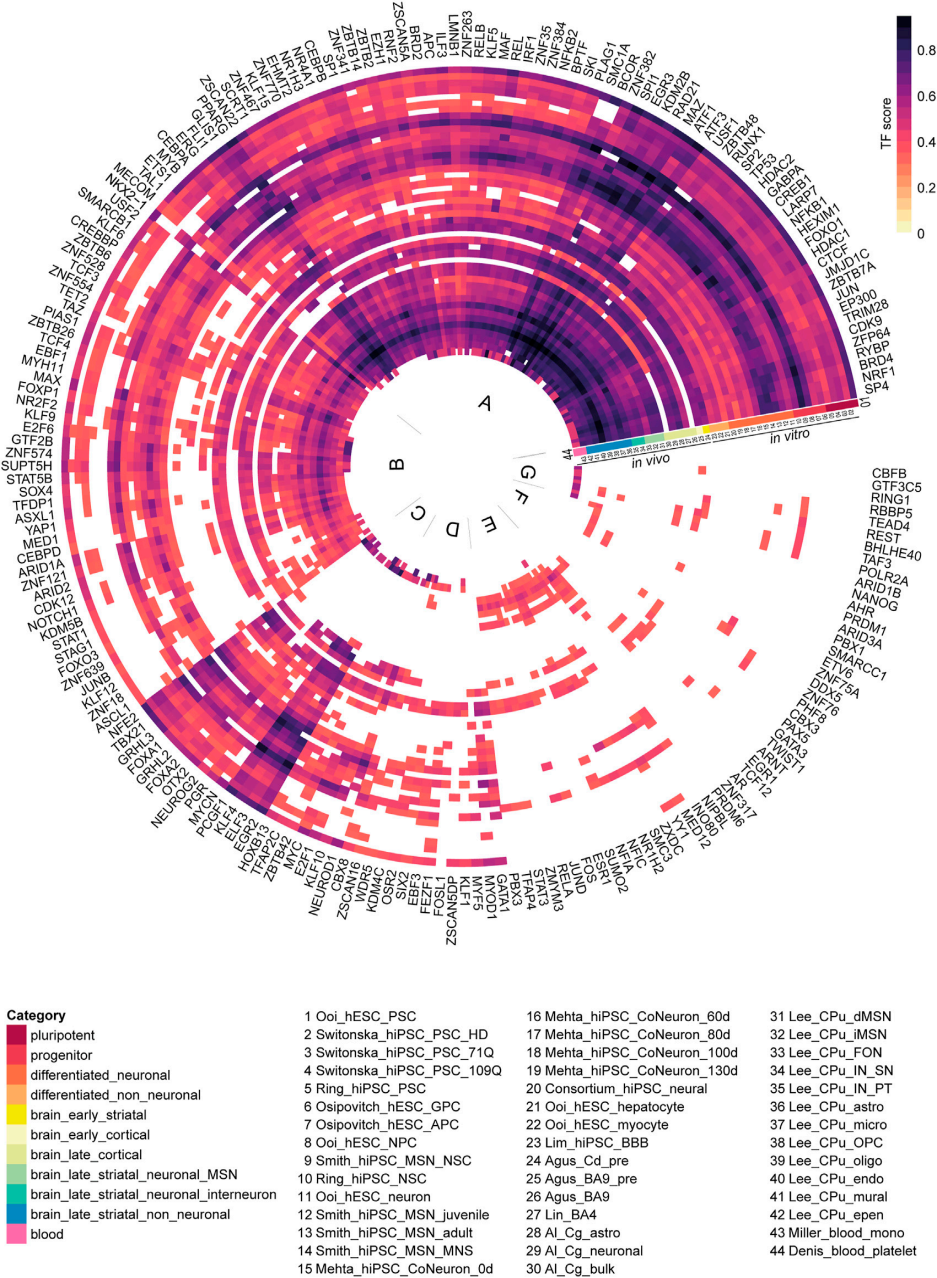
Potential enriched regulators for all 44 human differentially expressed gene (DEG) datasets. Each ring is a human DEG dataset, each spoke is a regulator. The regulator activity score was defined as the ratio of DEGs controlled by a regulator to the total number of DEGs in a dataset. For each dataset, regulators with an activity score higher than 0.3 were shown (*n* = 205). The regulators were clustered using the Ward D2 method. Based on commonality in the 44 human DEG datasets, regulators can be divided into 7 groups (A to G). Abbreviations: CPu, caudate putamen; astro, astrocyte; epen, ependymal; endo, endothelial; IN, interneuron; PT, Pvalb/Th-expressing; MSN, medium spiny neuron; MNS, MAP2+ and NES/SOX2-; OPC, oligodendrocyte progenitor cell; oligo, oligodendrocyte; micro, microglia; NSC, neural stem cell; Cg, cingulate cortex; FON, Foxp2/Olfm3-expressing neuron; SN, Sst/Npy-expressing; dSPN/iSPN, direct/indirect pathway spiny projection neuron; mono, monocyte; NPC, neural progenitor cell; CoNeuron, cortical neuron; Cd, caudate nucleus; pre, presymptomatic; GPC, glial progenitor cell; APC, astrocyte precursor cell; BBB, blood brain barrier.

In the circular heatmap depicting the regulators and their activity scores (Figure 5, Supplementary Dataset S8), one third of the heatmap (indicated as group A) depicts the most common set of regulators across the categories and the datasets. In this set of most common regulators, we observed regulators such as*, SP4, NRF1, BRD4, RYBP, ZFP64, HDAC1, FOXO1, CREB1, NFKB1, BRD2*, *SP1, CTCF and JUN* some of which were previously implicated in HD pathology (De Souza et al., 2016; Dunah et al., 2002; Steffan et al., 2000). Gene ontology analysis of these transcription factors (TFs) showed significant enrichment of genes related to transcriptional misregulation in cancer, pathways in cancer, Huntington’s disease, longevity regulating pathway and cell cycle (Supplementary Dataset S5). Regulators grouped in B show strong scores for all except for differentiated_non_neuronal, Mehta_hiPSC_CoNeurons, pluripotent cell and presymptomatic brain. Some of the less common regulators (Figure 5, group C), such as *PCGF1, KLF4, ELF3* and *EGR2* showed strong scores in all categories except the datasets in the late striatal category from Lee et al., 2020. Interestingly, the DEGs from the blood platelet dataset from Denis et al. were enriched for a group of high-scoring TFs (Figure 5, group G), namely *POLR2A, TAF3, BHLHE40, REST, TEAD4, RBBP5, RING1, GTF3C5* and *CBFB*. Of note, to test the reliability of our method, we checked the overlap between transcription factors (TFs) from the available H3K27ac motif data from a previous publication (Consortium, 2017) and the potential regulators predicted by hTFtarget for the same study. We found a significant overlap of 39 regulators (*p*-value = 3.79e-10; Fisher’s exact test; Supplementary Dataset S8).

**FIGURE 6:**
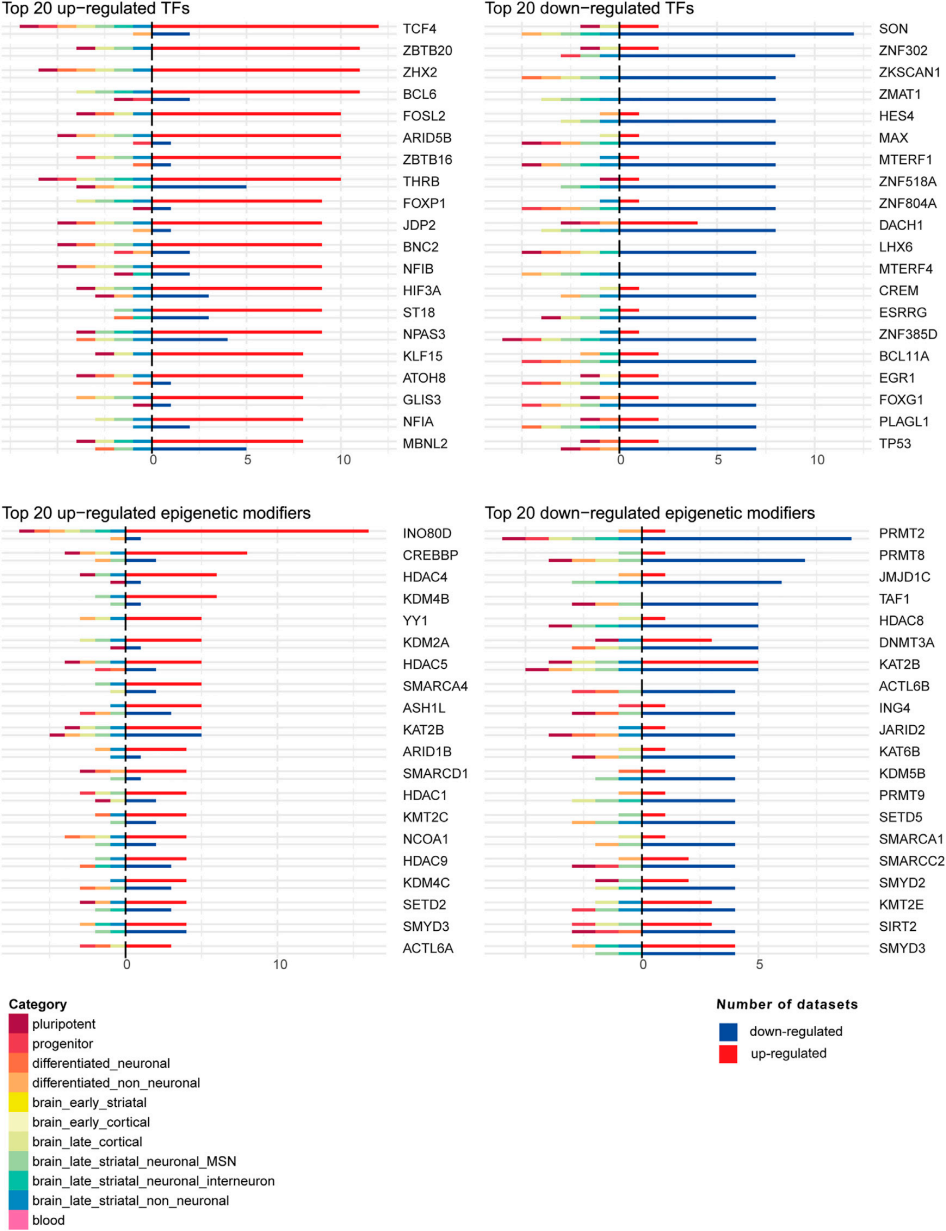
Top 20 up-regulated and down-regulated transcription factors (TFs) and epigenetic modifiers in the 44 human DEG datasets. For each TF or epigenetic modifier, shown were the numbers of datasets where the TF or epigenetic modifier was up-regulated or down-regulated. On the positive axis, the number of datasets in which the TF or epigenetic modifier is up-regulated or down-regulated are shown. On the negative axis, the category membership of the datasets where the TF or epigenetic modifier is up-regulated or down-regulated is indicated.

Next, we investigated the differential expression of TFs and epigenetic modifiers in HD using the human TFs (Lambert et al., 2018) and dbEM (Singh Nanda et al., 2016) databases, respectively, in each of the 44 datasets. Of 1,639 human TFs and 167 human epigenetic modifiers, we found differential expression of 1,388 TFs and 155 epigenetic modifiers in HD in at least one of the 44 datasets (Supplementary Dataset S9). Figure 6 shows the top common up-regulated and down-regulated TFs and epigenetic modifiers in HD in all 44 human datasets. The top common up-regulated TFs were *TCF4, ZBTB20, ZHX2* and *BCL6*, while the most common downregulated TFs were *SON, ZNF302, ZKSCAN1* and *ZMAT1.* The most common up-regulated epigenetic modifiers were *IN O 80D, CREBBP, HDAC4* and *KDM4B*, while the most common downregulated epigenetic modifiers were *PRMT2, PRMT8, JMJD1C* and *TAF1*. Interestingly, in MSNs, three of the top upregulated epigenetic modifiers, *KDM4B, KMT2C* and *SMARCD1* were uniquely downregulated, while the top downregulated epigenetic modifier genes *SETD5* and *PRMT8* were upregulated (Figure 6).

Finally, we investigated whether the predicted regulators of DEGs were themselves differentially expressed in HD in the 44 human DEG datasets. In total, 204 out of the predicted 557 regulators were differentially expressed in at least one of the datasets (Supplementary Dataset S9). Among the differentially expressed regulators, *TCF4, FOSL2, BCL6, ZBTB16, FOXP1, KLF15, RXRA, CUX1, CREBBP* and *NFIA* were the top upregulated regulators that were also predicted to be the regulators of DEGs in HDare, while the top downregulated ones were *ZKSCAN1, MAX, E2F3, BCL11A, EGR1, FOXG1, TP53, HMG20A, JMJD1C* and *STAT4* (Supplementary Dataset S9).

### Transcriptional Changes in Non-Human Models

In order to evaluate the suitability of non-human models to study transcriptional changes in HD patients, we compared the non-human data to human mRNA changes. For this, we retrieved 14 studies and 79 datasets (Table 5; Supplementary Figure S1). Among 14 studies, 12 were related to mouse models (Ng et al., 2013; Vashishtha et al., 2013; Crotti et al., 2014; Mielcarek et al., 2014; Achour et al., 2015; Langfelder et al., 2016; Miller et al., 2016; Handley et al., 2017; Jacobsen et al., 2017; Pan et al., 2018; Goodnight et al., 2019; Radulescu et al., 2019; Yildirim et al., 2019; Lee et al., 2020; Wertz et al., 2020), one study was related to sheep (Handley et al., 2017) and one to monkey (Goodnight et al., 2019).

**TABLE 5:** Characteristics of included non human HD studies. The table summarizes the PMID, sample, cell type, reference and key findings of each included non human study (Ng et al., 2013; Vashishtha et al., 2013; Crotti et al., 2014; Mielcarek et al., 2014; Langfelder et al., 2016; Handley et al., 2017; Jacobsen et al., 2017; Pan et al., 2018; Goodnight et al., 2019; Radulescu et al., 2019; Yildirim et al., 2019; Lee et al., 2020; Wertz et al., 2020).

PMID	Sample	Cell type	References	Key findings
25784504	R6/1 mouse	Striatum	Achour et al. (2015)	Found downregulation of neuronal identity genes accompanied by decrease in H3K27ac and RNAPII occupancy
31744868	R6/1 mouse; CHL2 mouse	Striatum	Yildirim et al. (2019)	Found abberant transcription and H3K27ac profiles at the presymptomatic stage and identified Elk-1 as a candidate early regulator
23872847	R6/2 mouse	Striatum, Cortex	Vashishtha et al. (2013)	Found reduced H3K4me3 levels at key neuronal genes and that inhibition of demethylases can ameliate pathology
25101683	R6/2 mouse; HdhQ150 mouse	Heart	Mielcarek et al. (2014)	Found heart pathology without mHTT aggregates or transcriptional dysregulation
28120936	R6/2 mouse	Cortex	Jacobsen et al. (2017)	Found significant expression of a fragment resulting from transgene integration in R6/2
32004439	R6/2 mouse	Striatum	Wertz et al. (2020)	Found mHTT toxicity modifiers such as Nme genes and several genes involved in methylation-dependent chromatin silencing and dopamin signaling
32681824	R6/2 mouse; HdhQ150 mouse	Striatum	Lee et al. (2020)	Found mtRNA release and upregulation of innate immune signaling in MSNs using cell type-specific transcriptomics
26900923	HdhQ175 mouse	Various tissues	Langfelder et al. (2016)	Found CAG length related gene modules such as MSN identity genes, cAMP signaling, cell death and protocadherin genes
31015293	BACHD mouse	Corpus Callosum	Radulescu et al. (2019)	Found that white matter abnormality precedes HD onset and that inhibition of mHTT in OPC ameliorates myelin pathology and HD-related behavioral deficits
29891550	Swiss Webster mouse	Primary Cortical Neuron	Pan et al. (2018)	Found that knockdown of Twist1 reverses expression of key neuronal genes and ameliorates HD pathology
23341638	E14 STHdhQ111 cell	Striatal Cell Line	Ng et al. (2013)	Found extensive changes in DNA methylation accompanying altered gene expression and identified AP-1 and SOX2 as transcriptional regulators for HD-related methylation
24584051	BV2 microglia cell	Cell Culture	Crotti et al. (2014)	Found cell-autonomous pro-inflammatory transcriptional activation in microglia driven by PU.1 and C/EBPs
31722751	Rhesus macaque PSC	PSC, NPC, Astrocyte	Goodnight et al. (2019)	Found downregulation of p53 signaling and cell cycle pathway in NPCs and upregulation in astrocytes perhaps driven by E2F
29229845	OVT73 sheep	Striatum	Handley et al. (2017)	Found upregulation of urea transporter SLC14A1 and other osmotic regulators in prodromal sheep striatum

Similar to human data, in the mouse data, we checked the expression changes of 45 previously published key HD genes (Seredenina and Luthi-Carter, 2012; Vashishtha et al., 2013). Among the 45 key HD genes, the commonly dysregulated genes in mice were *Ryr1, Scn4b, Gpr6, Hrh3, Foxp1, Gpr88, Rgs9, Pde10a, Arpp21, Adora 2a, Adcy5, Drd2, Pcp4* (Supplementary Figure S6A). Moreover, the available monkey dataset showed genes such as *CAMKV, KCNIP2, OPRD1, RGS9, HRH3, ADORA2A, KLF16, CNR1, OPRK1, FOXP1, NR4A1, TESC, GSN* and *RARB* that were dysregulated in monkey model of HD (Supplementary Figure S6B). These findings provide evidence that the HD animal models faithfully recapitulate human HD regarding its molecular pathology.

**FIGURE 7:**
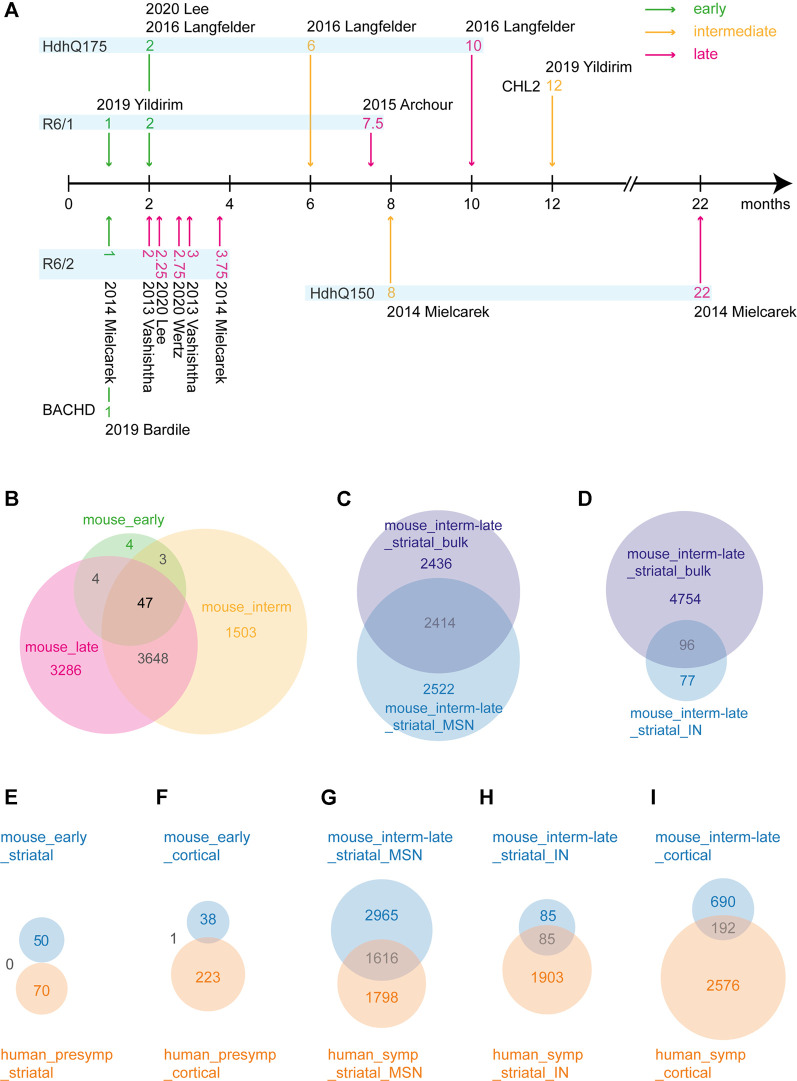
Differentially expressed gene (DEG) analysis and overlap between mouse and human datasets. **(A)** HD mouse model disease progression timeline. Based on existing literature, timepoints from HD models are categorized as early, intermediate and late disease stage. **(B)** Venn diagram of DEG overlap between mouse early, intermediate and late categories. **(C)** Venn diagram of DEG overlap between mouse intermediate/late bulk striata and mouse intermediate/late striatal medium spiny neurons. **(D)** Venn diagram of DEG overlap between mouse intermediate/late bulk striata and mouse intermediate/late striatal interneurons. **(E–I)** Corresponding cell types and disease stages between human and mouse categories were compared. Blue denotes mouse categories; orange denotes human categories.

While different HD mouse models show disease progression at different ages, we sorted all the datasets along a disease progression timeline based on the literature regarding HD mouse models (see methods). For this, three studies were excluded as they were cell culture models and could not be confidently assigned to a disease stage. Then, we categorized the mouse datasets as early, intermediate and late stages (Figure 7A), in which the latter two were comparable to the symptomatic stage in humans. Out of 1,037, 13788 and 12609 total DEGs (Supplementary Dataset S10), 58, 5201 and 6985 genes were frequently dysregulated in early, intermediate and late disease stage of HD mice, respectively, of which only 47 genes were common across all the three disease stages (Figure 7B). Some of the common genes were *Scn4b, Penk, Arpp21, Kcnab1, Ngef, Ppp4r4, Gpx6, Slc4a11, Rgs4, Rgs14, Adora2a, Gpr153, Plk5* and *Wnt4* (Supplementary Dataset S10). In early stage, genes such as *Clspn, Gpr153, Gpx6, Krt9, Penk* and *Plk5* were frequently dysregulated, while in intermediate stage, *Penk, Atp2b1, Scn4b, Pde10a* and *Rbfox1* were dysregulated to name a few. In late stage, *Pcp4, Scn4b, Pde10a, Penk Atp2b1, Hspa8* and *Nrxn3* were dysregulated among many others (Supplementary Dataset S10). The top upregulated and downregulated genes in each dataset are shown in Table 6. Additionally, we compared single cell transcriptomic analysis of medium spiny neurons and interneurons with bulk striatal data. We observed that of 4,936 differentially expressed genes in MSNs, 2,522 genes were unique in medium spiny neurons and out of 173 in interneurons, 77 genes were unique to interneurons (Figures 7C,D).

**TABLE 6:** Mouse top differentially expressed genes (DEGs) in each category. The table summarizes the top DEGs in brain subcategories in mouse, first ranked by the frequency of DEGs, then by the average FDR value of DEGs across all datasets in that subcategory.

Category	Top 20 DEGs
Early	Plk5, Krt9, Clspn, Gpx6, Penk, Gpr153, Pomc, Tmprss6, Gpd1, Neto2, Rgs4, Cnr1, Ddit4l, Wnt4, Ppp4r4, Adora2a, Aqp1, Traip, Sec14l3, Plekhg5
Intermediate	Penk, Scn4b, Rbfox1, Pde10a, Ttyh1, Ppp1r1b, Gpm6b, Arpp21, Atp2b1, Ypel2, Polr2a, Ptprd, Plp1, Kcnq5, Rgs9, Phactr1, Gsg1l, Pcdh11x, Scaper, Pcp4
Late	Pcp4, Scn4b, Penk, Pde10a, Nrxn3, Atp2b1, Hspa8, Ttr, Phactr1, Ppp3ca, Arpp21, Kcnab1, Spag5, Sec14l1, Arpp19, Nlgn1, Ckb, Kcnq5, Slc4a4, Ablim2
Early_cortical	Serpina1c, Alb, Mup3, Grk4, Gc, Pomc, Fgg, Apob, Gpr153, Serpina1b, Mup20, Ces3a, 2810007J24Rik, Mup10, Gad1, Rerg, Tat, Cyp1a2, Cyp2c50, Serpina1d
Interm-late_cortical	Ddit4l, Gucy2g, Tnnc1, Hapln1, Luzp2, Irf7, Otop2, Gad1, Rgs20, Gfra2, Scube1, Scn4b, Vip, Slc39a10, Glt8d2, Gpr83, Car12, Stard8, Gpr153, Kcna1
Early_striatal	Plk5, Krt9, Clspn, Gpx6, Penk, Tmprss6, Gpd1, Neto2, Rgs4, Cnr1, Ddit4l, Wnt4, Ppp4r4, Adora2a, Aqp1, Traip, Sec14l3, Id4, Slc4a11, Egr2
Interm-late_striatal_MSN	Pde10a, Osbpl8, Pde1b, Gpm6b, Plcb1, Phactr1, Atp2b1, Kcnab1, Kctd1, Homer1, Scn4b, Inf2, Baiap2, Pde7b, Adcy5, Penk, Rbfox1, Rgs9, Ppp4r4, Rhobtb2
Interm-late_striatal_interneuron	Penk, Pde10a, Nlgn1, Osbpl8, Pcdh9, Atad2b, Cttnbp2, Brinp3, Cnr1, Ttr, Pde4b, Galntl6, Clstn2, Ptprs, Kcnip4, Fam155a, Csmd2, Caln1, Snhg11, Erc2
Interm-late_striatal_bulk	Ddit4l, Gpx6, Arpp19, Abi3bp, Scn4b, Ryr1, Gpr83, Rgs4, Cd4, Gpr88, Kcnab1, Pde10a, Pcp4, Clec12a, Rgs9, Adora2a, Adcy5, Chrm4, Krt9, Drd2
Brain	Scn4b, Penk, Kcnab1, Pde10a, Arpp21, Neto2, Pcp4, Sec14l1, Ryr1, Pde7b, Ppp1r1a, Ngef, Arpp19, Rgs9, Cnr1, Rhobtb2, Kcnq5, Pmepa1, Itpka, Ppp1r1b

To evaluate how well the mouse models recapitulate the transcriptional changes in human HD, we compared early disease stages in mice with presymptomatic human brain, and intermediate and late stages in mice with symptomatic human brain. In early/presymptomatic stages, we observed no overlapping DEGs in the striatum and only one overlapping DEG, *Serpina1* in the cortex (Figures 7E,F, Supplementary Dataset S10). The overlap between the mouse and human data was comparably large with 1,616, 85 and 192 overlapping DEGs between intermediate-late mouse MSNs, interneurons and cortex with corresponding symptomatic HD human brain subcategories (Figures 7G–I). Some of the genes common between human and mouse are *Mtf2, Mtus2, Slc25A, Smarcal1, Snap25, Smarca2, Mt-co3, Camk4* (Supplementary Dataset S10). Moreover, the expression changes of the three common heat shock genes from the human subcategories, *Dnajb1, Hspa1b* and *Hspb1,* were variable in the mouse datasets. While the expression of *Hspa1b* and *Hspb1* weren’t altered in majority of the striatal mouse datasets, *Dnajb1* showed upregulation in some datasets and downregulation in some others in HD mice Table 4).

### Comparison of mRNA Changes to Previous Microarray Studies

We also compared the mRNA data included in our analysis with two key microarray studies in the field, Hodges et al. (2006) and Kuhn et al. (2007). As reported in Hodges et al. (2006), we observed the majority of dysregulated genes in caudate or striatum, which they showed in their study to be independent of neuronal loss in this area. Out of the listed top 30 upregulated and down regulated genes, 15 and 20 genes, respectively were common in our data (Supplementary Dataset S6). Dysregulation of genes associated mainly with heat shock proteins, chaperonins, protein folding and response to unfolded protein were in consistency with the microarray study in human HD caudate. In accordance with Hodges et al. (2006), we also report that the dysregulated genes in MSNs are mostly associated with neurotransmitter receptor activity, as well as with those conveying signals from excitatory amino acids like glutamate receptor signalling pathway. Furthermore, Kuhn et al. (2007) demonstrated that the different genetic models of mouse could replicate the transcriptomic changes of human HD caudate, however, regional specificity in most mouse models was considered to be very low. The study showed stronger similarity in downregulated genes between mouse models and human HD. We observed in our study 43 genes out of 60 top mouse genes that were concordant with human caudate in the microarray analysis of Kuhn et al., 2007. Some of the common genes between our study and Kuhn et al. (2007) are *RASGRP2, RGS14, HOMER1, PENK, PPP3CA, PLCB1, PPP1R1A, CAMK2A* and *RGS4* (Supplementary Dataset S6). Overall, our finding of genes dysregulated in human and non-human HD models using RNA-seq were consistent with the findings from previous key microarray studies.

## Discussion

In Huntington’s disease, transcriptional dysregulation is one of the early and central contributors to the disease pathogenesis. Examination of these changes by comparing gene expression patterns, their regulators and the affected pathways is therefore crucial for understanding the pathophysiology of the disease. Although there are numerous studies, including both research and review work, focusing on gene expression changes in HD, a systematic review of this literature has been lacking. Here, we performed a comprehensive systematic review of all available transcriptional profiling studies in HD that used RNA sequencing technique, published by the time of our screening (August 2020). Our systematic review protocol has been registered at the Open Science Framework (OSF) (Chasapopoulou et al., 2020). We retrieved literature from PubMed and gene expression databases from BioProject, ArrayExpress, ENA and EGA following the PRISMA guidelines (Supplementary Figure S1). After excluding papers and datasets that did not meet the inclusion criteria (Figure 1), we grouped the human studies based on tissue/cell types and disease stages (Figure 2) for the assessment of differential expression of genes as well as gene ontology and gene set enrichment analyses in cell culture models, postmortem brain and blood samples from HD patients. Further, we examined transcription factors and epigenetic regulators, which may underlie the observed transcriptional dysregulation in HD. Additionally, we systematically reviewed non-human HD studies and compared differential gene expression patterns in mouse models to the human data.

In human studies, comparison of the most frequent DEGs across subcategories revealed that human HD cell culture models were distinct in their transcriptional profiles compared to postmortem brain samples, suggesting that the cell culture models may not fully recapitulate the molecular pathology in human HD (Figure 3A, Supplementary Figure S3). In cell culture models, *IGDCC3* and *XKR4* genes were commonly dysregulated in all studies. *IGDCC3* takes part in nervous system development (Salbaum, 1998) and *XKR4* is involved in apoptotic processes during development (Suzuki et al., 2014). On the other hand, the most commonly dysregulated genes in post-mortem brain samples were *DNAJB1*, *HSPA1B* and *HSPB1* that were reported in all postmortem datasets except for the MSN subcategory, while *HSPH1* and *SAT1* were reported in all symptomatic subcategories. *SAT1* is involved in p53-mediated ferroptotic responses and tumorigenic aggressiveness (Kang et al., 2019), while the other genes are involved in heat shock response. Heat shock response is initiated during cellular stress that leads to regulation of heat shock proteins. Differential expression of these genes are in consistency with the reports of dysregulation of heat shock genes in HD in many studies (Gomez-Paredes et al., 2021; Labbadia et al., 2011; Labbadia and Morimoto, 2013; San Gil et al., 2017). Importantly, several heat shock proteins have been reported to be associated with regulation of huntingtin aggregation (Orozco-Diaz et al., 2019), including *DNAJB1, HSPA1A, HSPA1B* and *HSPB1* that were reported to suppress polyglutamine aggregation in mammalian cells (Gomez-Paredes et al., 2021; Hay et al., 2004; Zourlidou et al., 2007). In our study, we observed mostly an upregulation of *DNAJB1, HSPA1B* and *HSPB1* in HD. In presymptomatic postmortem brain, the upregulation was even higher in comparison to the symptomatic postmortem brain regions, which could be a mechanism of coping with protein misfolding during early stage of the disease. In contrast, MSNs did not show upregulation of heat shock response genes in the symptomatic stage of HD, which might implicate a lack of protein quality control and stress response leading to enhanced vulnerability of these neurons in HD. Importantly, while mouse datasets showed diverse changes of expression of *Dnajb1, Hspa1b* and *Hspb1* were not differentially expressed in majority of the striatal mouse datasets, indicating consistency with the findings from the caudate data in human.

Protein misfolding is one of the hallmarks in not only HD but also is associated with other neurodegenerative disorders like Alzheimer’s Disease (AD), Parkinson’s Disease (PD) and amyotrophic lateral sclerosis (ALS). Ruffinni et al. (2020) have identified an overlap of 139 genes among HD, AD, PD and ALS, which were also involved in the response to heat and hypoxia (Ruffini et al., 2020). In this context, modulation of heat shock response geneshas also been proposed to be a potential therapeutic target in neurodegenerative diseases associated with misfolded protein aggregates (Ramos et al., 2018; Gomez-Paredes et al., 2021; Labbadia and Morimoto, 2013). *Hsp 27* and *Hsp 70* overexpression were shown to exert protective effects in cell culture models. Contrastingly, overexpression of *Hsp27* and *Hsp 70* in R6/2 mice could not improve the disease phenotype. In acute conditions such as kainate-induced or ischemic injury, *in vivo* studies also reported to show protective effects, suggesting difference in heat shock response in acute and chronic disease conditions (Hay et al., 2004; Zourlidou et al., 2007; Labbadia et al., 2011). Labbadia et al., 2011 demonstrated a transient positive effect on disease phenotype via pharmacological activation of HSF1, a major regulator of heat shock response, in HD mice. As potential explanation for this transient effect, the authors suggested that as the disease progresses, the progressive reduction in histone acetylation may lead to reduced HSF1 binding, consequently reducing the expression of heat shock response genes. Hence, a combinatorial targeting approach targeting both the heat shock response regulators and the altered epigenome could be considered in future studies. In this line, we previously reported highly coordinated chromatin and transcriptional changes in the brains of HD mice, revealing the epigenomic changes as strong candidates for targeting transcriptional dysregulation in HD (Vashishtha et al., 2013; Yildirim et al., 2019). Moreover, all late brain categories shared the Spermidine/Spermine N1-Acetyltransferase 1 (*SAT1*) gene, which has been reported to have positive influence on recognition and memory in a rodent model of HD (Velloso et al., 2009). Vivo et al., 2001 reported that during severe brain atrophy, the concentration of spermine reduces in neurodegenerative diseases like Parkinson’s disease and HD and in the aging brain (Vivo et al., 2001). Additionally, spermidine pathway was shown to modulate muscarinic and NMDA receptor blockade in hippocampus, preventing reactive astrogliosis and mnemonic deficits in HD (Velloso et al., 2009). In MSNs, neurodegeneration might influence *SAT1 gene* expression leading to spermine depletion-associated gliosis and mnemonic deficits. In our study, we observed upregulation of the *SAT1 gene* in the presymptomatic brain, and downregulation in most of the symptomatic brain regions, specifically in MSNs (Supplementary Dataset S4).

Specifically focusing on iMSNs, the most vulnerable cell types to mutant *HTT* toxicity, we observed that dMSNs and Foxp2/Olfm3-expressing neurons (FON) share most genes with iMSNs. In contrast, only a few genes were in common with blood monocytes, platelets as well as with presymptomatic BA9 and caudate nucleus (Figure 3B). Furthermore, differential expression of genes associated to dysregulation of mitochondrial complex I, cytochrome c oxidase, ATP synthase and kinases were observed in iMSNs. Regarding mitochondrial genes, we observed that *MT-ND3, MT-CO2, MT-ND4L, MT-ND4, MT-ATP6, MT-CO3, SLC26A3, INO80D, MT-CO1* and *MT-ND* genes were upregulated in iMSNs in HD. Impaired energy metabolism due to mitochondrial dysfunction is considered as one of the contributing factors in HD as well as in other neurodegenerative diseases (Gomez-Paredes et al., 2021; Labbadia and Morimoto, 2013). Lee et al. (2020) also reported mitochondrial genes to be the most upregulated genes in iMSNs in HD. In HD, mitochondrial dysfunction is known to cause mtRNA release, which subsequently triggers innate immune signaling leading to enhanced vulnerability of iMSNs (Lee et al., 2020).

**FIGURE 8:**
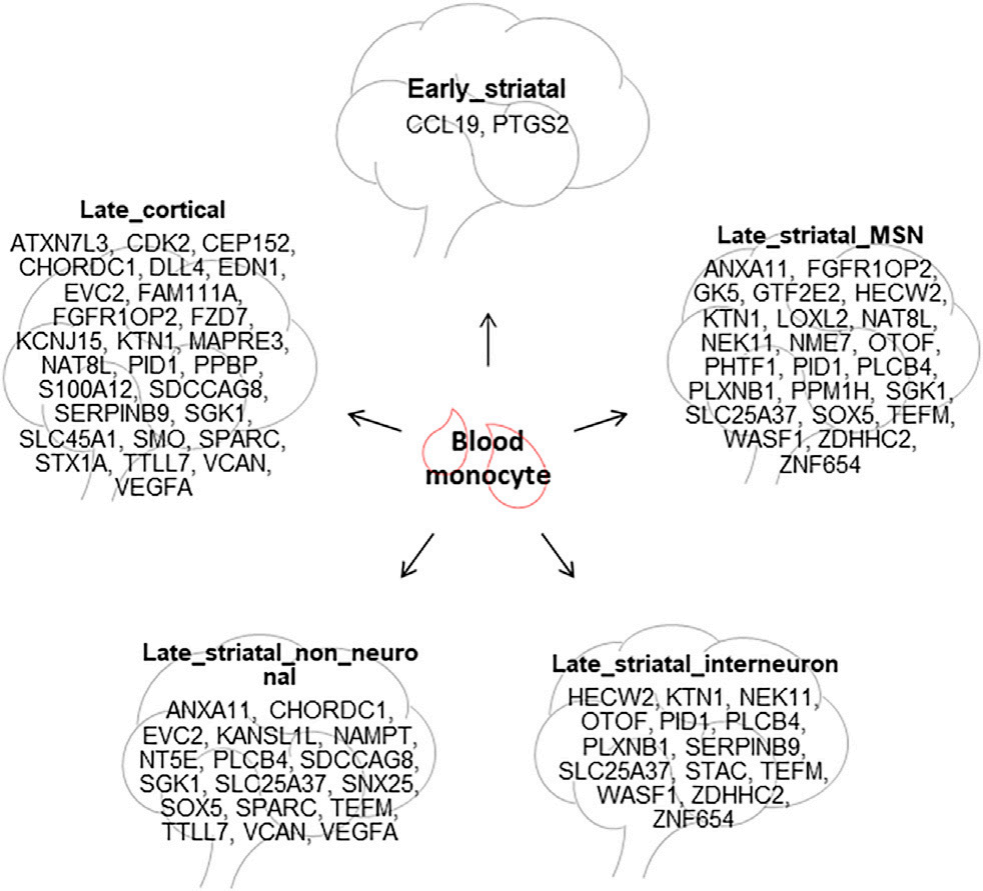
Potential blood biomarkers for HD. The common DEGs between HD blood monocytes and different HD brain tissues are summarized.

Blood is of great interest, since it can provide accessible diagnostic and prognostic biomarkers (Borovecki et al., 2005). In this study, we found that the blood had no common DEGs with presymptomatic BA9 and only two common DEGs with presymptomatic caudate, which is in line with the fact that the blood samples in the included datasets were obtained from symptomatic HD patients. The number of common DEGs was significantly more between blood and symptomatic brain regions, for instance, 50 genes with BA9, 25 genes with iMSN, 23 genes with FON, 22 genes with dMSN, and 20 genes with astrocytes (Figure 3C). These concordant gene expression changes could be further investigated to explore their potential as biomarkers in HD (Figure 8).

Our gene set enrichment analysis results showed that the human HD datasets were commonly enriched for many gene sets such as dysregulation of protein translation and targeting, heat shock response, immune response, transcription regulatory region DNA-binding, cAMP signaling, synaptic transmission, signaling pathway and p53 pathway (Figure 4). On the other hand, there were also different enrichments depending on the categories. While the cellular models showed enrichment of cellular processes and developmental pathways, postmortem brains showed distinct enrichment of major gene sets related to protein folding, translation and localization, immune response, apoptotic processes, stress responses and synaptic transmission. Of note, in line with previous studies (Seredenina and Luthi-Carter, 2012; Vashishtha et al., 2013), some of the key HD genes including *PCP4, RARB, NGEF, ADCY5, CAMKV, GRM3, BCL11B, CNR1, RGS9, DRD2* and *CAMKK2* were downregulated in postmortem neuronal cells but upregulated in some of the cell culture models (Supplementary Figure S4), indicating the distinct differential gene expression patterns in the postmortem primary tissues and cellular models in HD. In blood, gene set enrichment analysis showed that signaling pathways associated with IL-17, *JAK-STAT* and cancer related gene sets were enriched.

Furthermore, to search for key regulators underlying the transcriptional dysregulation in HD, we examined both the regulators predicted by DEGs and regulators that were differentially expressed themselves in HD. In the first analysis, we found potential regulators like *SP4, NRF1, BRD4, RYBP, ZFP64, HDAC1, FOXO1, CREB1, NFKB1, BRD2*, *SP1 and CTCF* that were common in all subcategories and regulators predicted only in specific categories such as *TCF12, AR, ZNF317 and PRDM6* in brain_late_striatal_non_neuronal subcategory *and CBFB, GTF3C5, RING1, RBBP5, TEAD4 and BHLHE40* in blood (Figure 5). The second analysis uncovered many differentially expressed transcription factors and epigenetic regulators, some of which were indicated as potential master regulators in HD by previous studies, e.g., *JUN* related transcription factors (Perrin et al., 2009), *FOS* related transcription factors (Cabanas et al., 2017), *CBP* (Giralt et al., 2012)*, EGR1* (Chandrasekaran and Bonchev, 2016) and *ELK1* (Yildirim et al., 2019). Additionally, Valor et al., reviewed the role of *SP1, CREB, NF-KB, P53, REST, HIPPI, FOXP1* as well as *HSF1* in HD pathology (Valor, 2014). Among these, binding of HSF1 to their loci was observed to be depleted in in vitro HD studies. *In vivo*, both the expression of heat shock genes as well as HSF1 binding were reported to be modulated in HD (Valor, 2015). Among the top upregulated and downregulated transcription factors and epigenetic regulators (Figure 6), *TCF4* is involved in regulation of synaptic plasticity, DNA methylation and memory function (Kennedy et al., 2016), *SON* is known to participate in transcription, pre-messenger RNA splicing and cell cycle regulation (Lu et al., 2014), *INO80* complex an ATP-dependent nucleosome remodeling complex conserved from yeast to human (Jin et al., 2005) and *PRMT2* an arginine methyltransferase and a coactivator of the androgen- and estrogen-mediated transactivation (Meyer et al., 2007). Interestingly, some of the top upregulated epigenetic modifiers, *KDM4B, SMARCD1* and *KMT2C* were downregulated and two of the top downregulated epigenetic modifiers, *PRMT8* and *SETD5* were upregulated in MSNs (Figure 6). Moreover, there were some regulators, such as *TCF4, FOSL2, BCL6, RXRA, CUX1, CREBBP, E2F3, BCL11A, EGR1, FOXG1, TP53, JMJD1C* and *STAT4*, which were both changed in expression and were predicted to be the regulators of DEGs in HD (Supplementary Dataset S9).

In non-human models, we observed some of the well reported DEGs in HD such as, *Adora2a, Adcy5, CamkV and Penk* (Seredenina and Luthi-Carter, 2012; Vashishtha et al., 2013; Shao et al., 2020)*.* In particular, the mouse data showed relatively fewer dysregulated transcripts in the early stages, which expanded tremendously during the progression of the disease at later stages (Figure 7B). We found a large concordance of transcriptional changes between the mouse and human data in the progressive disease phase, which indicates the suitability of mouse models for studying HD molecular pathology. Notably, we found that only around half of the genes were common between MSNs data and the whole striatum data in mouse models (Figure 7C). This is likely due to the contribution of other cell types of the striatum to the bulk data. Also, differences between single cell RNA-seq and bulk RNA-seq should be taken into consideration. In this context, limitations of the present study should be acknowledged. We encountered technical challenges dealing with methodological variability between datasets. As the scope of our study was not the re-analysis of RNA sequencing data, we used the DEGs and the backgrounds generated by the authors, using the filtering and cutoffs indicated in the respective papers. Some of the studies applied single-cell transcriptomics, while others used bulk tissue transcriptomics. Although, single cell RNA-seq data tended to have larger numbers of significant DEGs, they were treated similarly with bulk RNA-seq data. Hence, these differences in study settings, techniques and data analysis in each paper may have generated potential bias in the analysis of our study.

**FIGURE 9:**
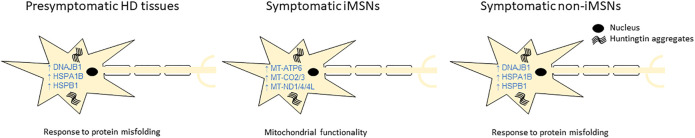
Key differentially expressed genes (DEGs) and pathways potentially responsible for HD pathology. The diagram summarizes the key DEGs and pathways generated from data synthesis, which may explain the differential vulnerability of indirect pathway medium spiny neurons (iMSNs) compared to other cell types in brain.

Nevertheless, in this study, we were able to show that MSNs exhibit unique dysregulation of genes in comparison to the other brain regions. Except for MSNs, all cortex and striatum categories shared three heat shock response and unfolded protein response genes, *DNAJB1*, *HSPA1B* and *HSPB1,* that were upregulated in both early and late stages of HD (Figure 9). In iMSNs, we found unique dysregulation of mitochondrial function related genes (Figure 9). Additionally, downregulation of *SAT1* gene may be related to early neurodegeneration of MSNs, which might also be the consequence of unique dysregulation of mitochondrial function related genes. We also found differential expression and predicted activity of a number of transcription factors such as *BCL11A, BCL6, EGR1* and *FOSL2,* and epigenetic regulators like *CREBBP, HDAC1, KAT2B, KDM4C* and *KDM5B* in human HD. This review hence summarizes and confirms reports from previous studies as well as presents new observations by systematically reviewing all the literature on transcriptional analysis using RNA sequencing in HD.

## Data Availability

The datasets presented in this study can be found in online repositories. The names of the repository/repositories and accession number(s) can be found in the article/Supplementary Material.
