# Supplementary figures and images for: A Systematic Review of Transcriptional Dysregulation in Huntington’s Disease Studied by RNA Sequencing
Source: Front Genet. 2021 Oct 15;12:751033. doi: 10.3389/fgene.2021.751033 (PMC8554124; doi:10.3389/fgene.2021.751033)

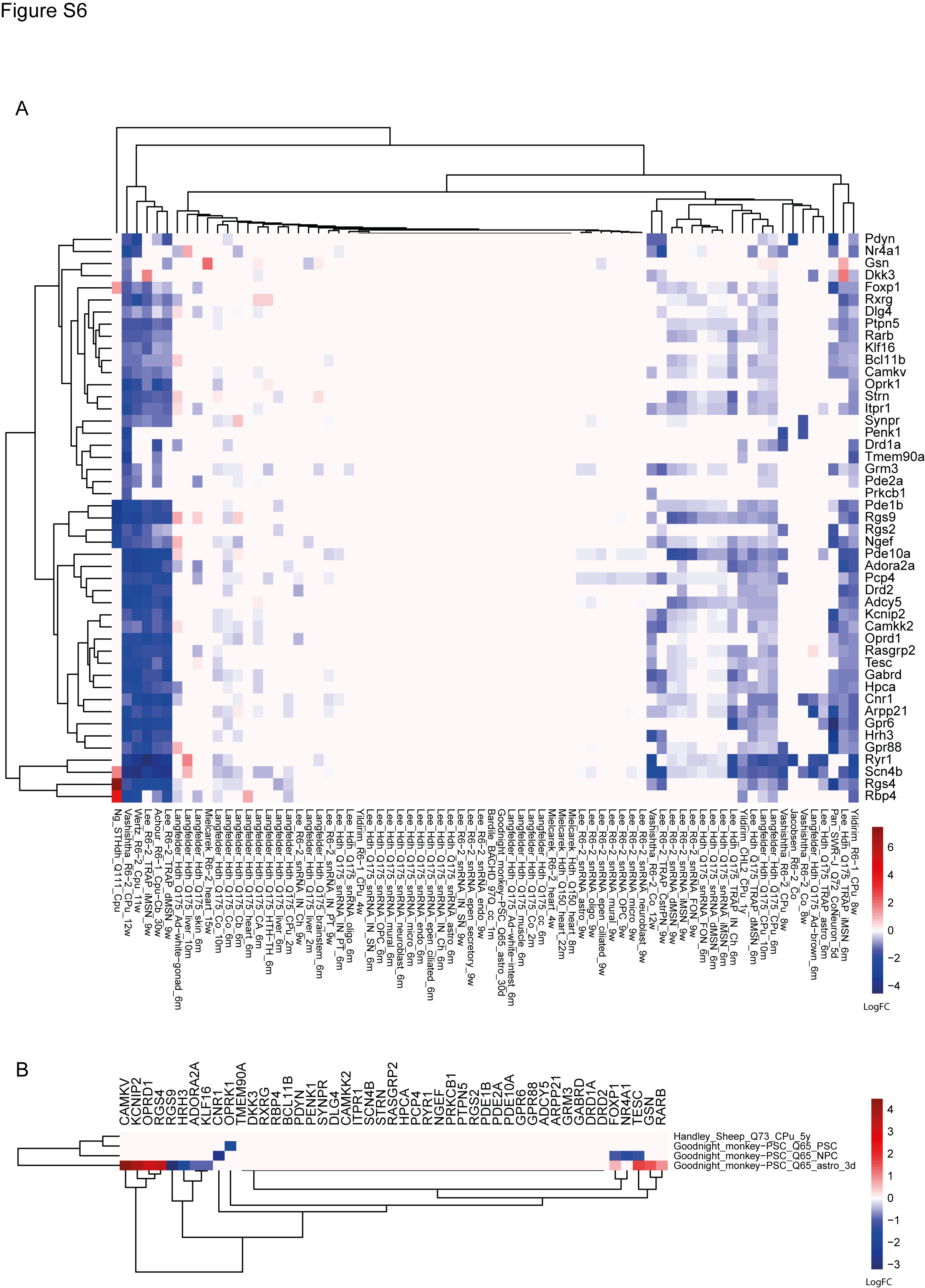

Supplement: Supplementary file 2 [file Image6.TIF]

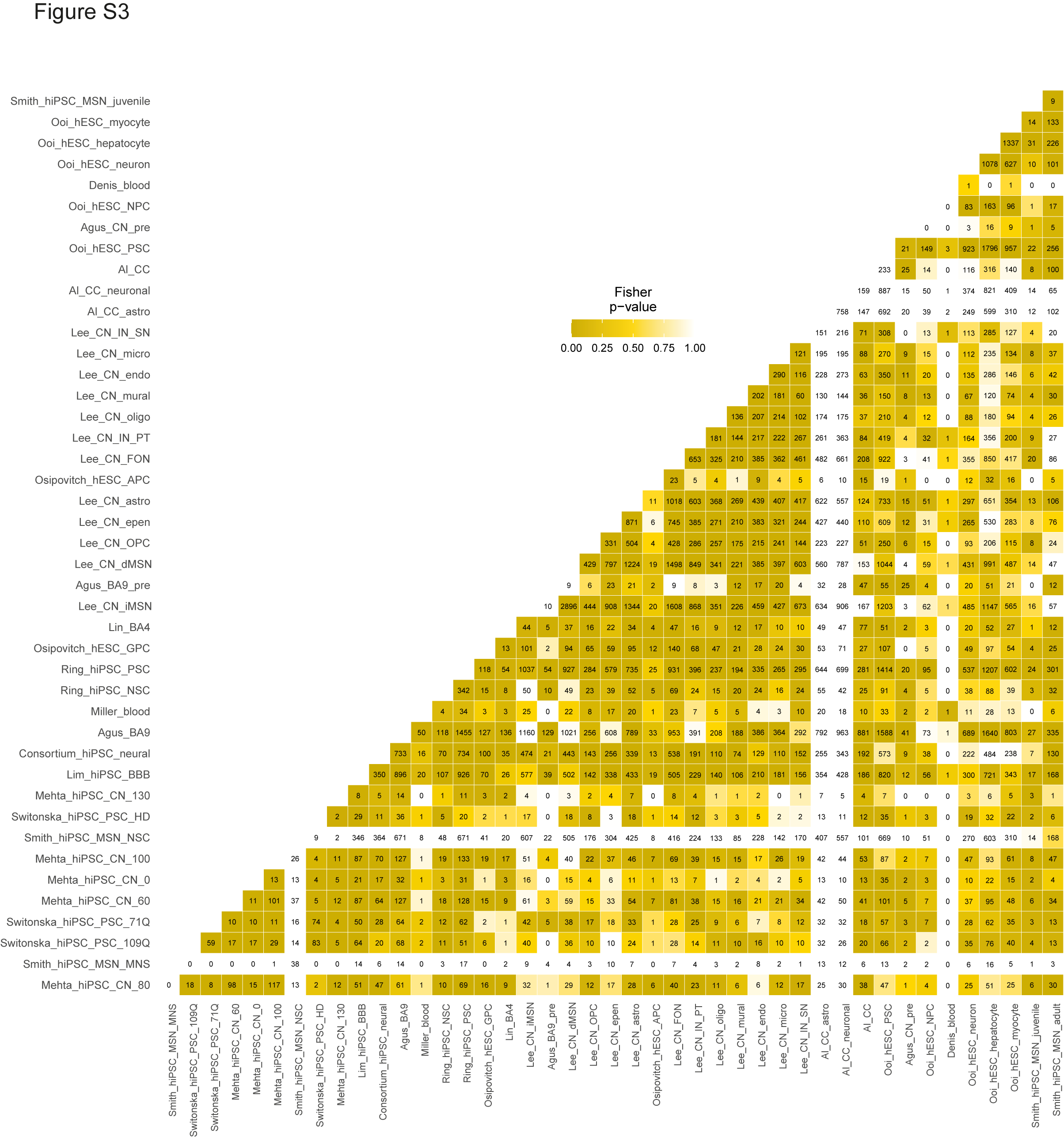

Supplement: Supplementary file 4 [file Image3.TIF]

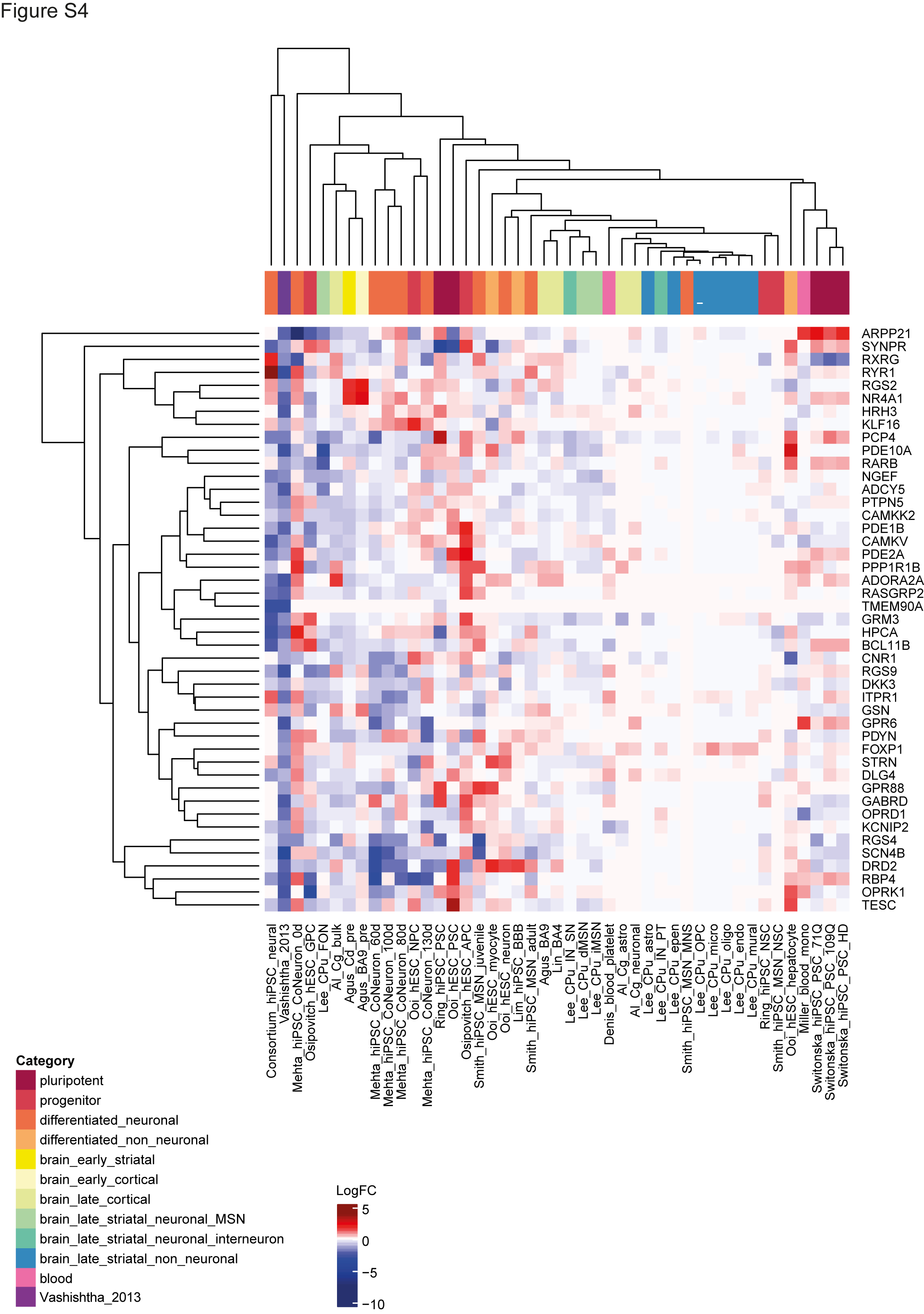

Supplement: Supplementary file 6 [file Image4.TIF]

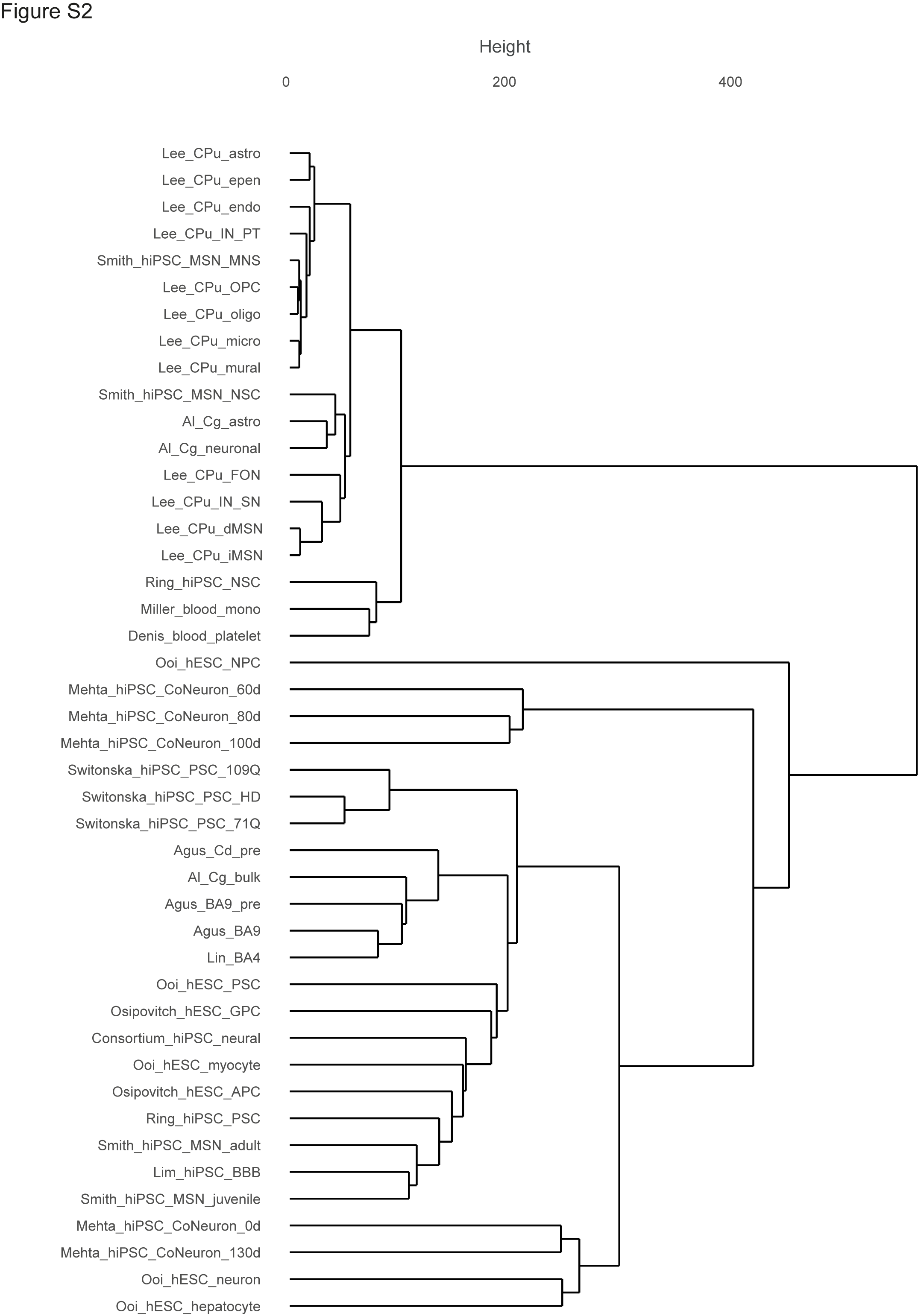

Supplement: Supplementary file 7 [file Image2.TIF]

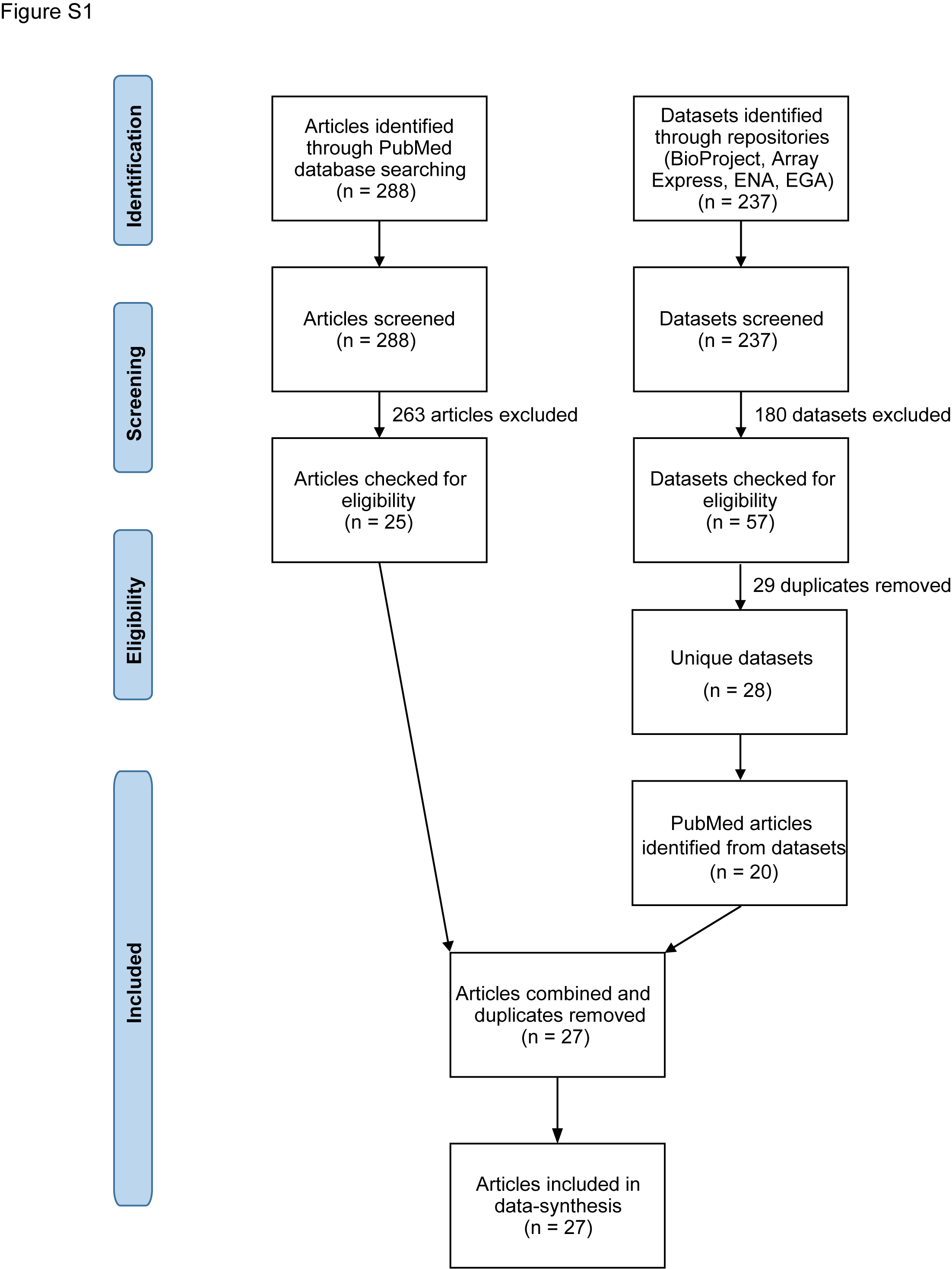

Supplement: Supplementary file 9 [file Image1.TIF]

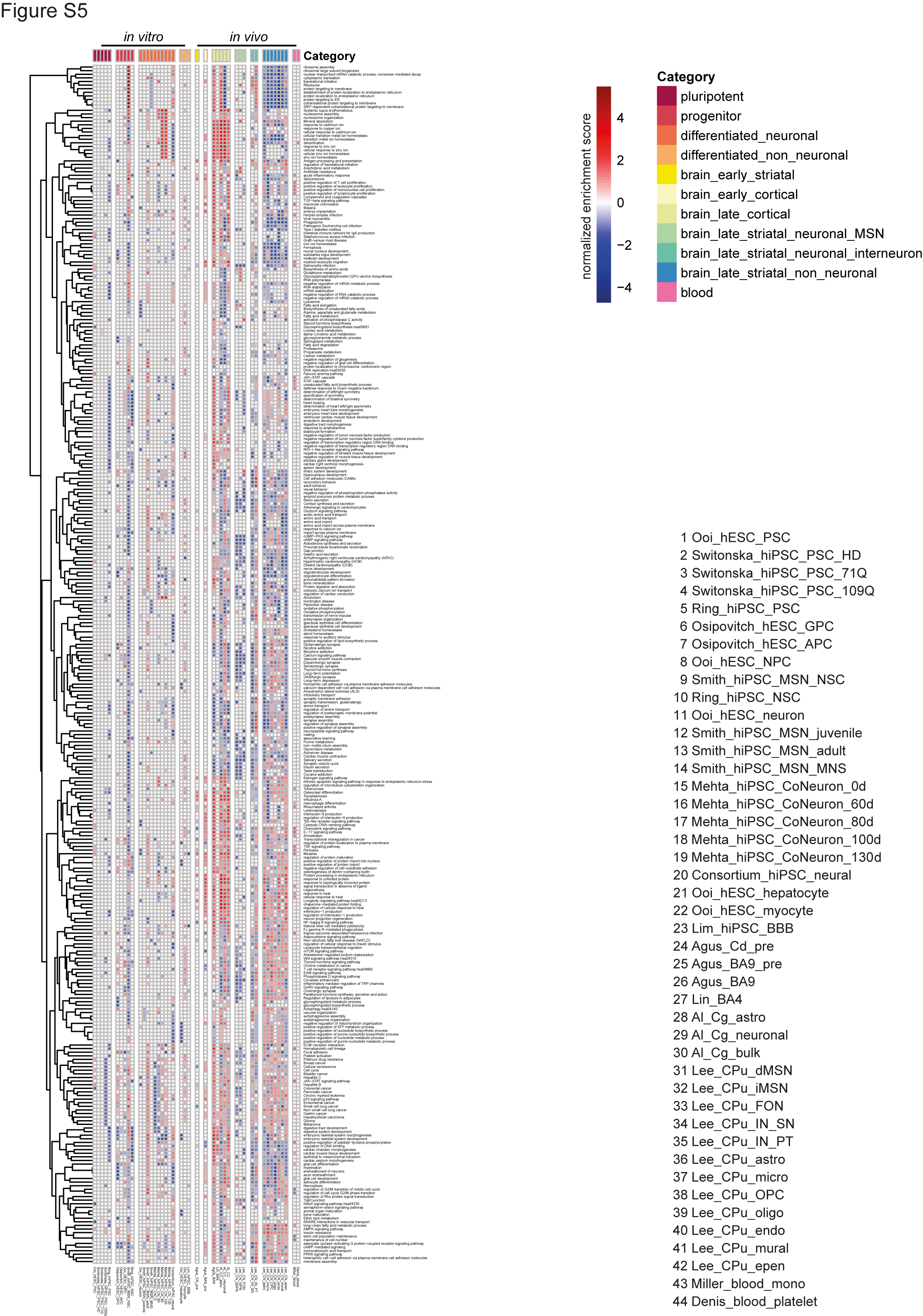

Supplement: Supplementary file 14 [file Image5.TIF]
