# Supplementary material for: A Systematic Review of Transcriptional Dysregulation in Huntington’s Disease Studied by RNA Sequencing
Source: Front Genet. 2021 Oct 15;12:751033. doi: 10.3389/fgene.2021.751033 (PMC8554124; doi:10.3389/fgene.2021.751033)
Supplement: Supplementary file 15 [file DataSheet11.docx]

***Supplementary Material***

**Supplementary Figures**

**Figure S1. PRISMA flow diagram of the HD systematic review.** The number of articles or datasets that remained after each phase of the screening is indicated in the box.

**Figure S2. Hierarchical clustering dendrogram of 44 human HD datasets.** 44 human HD datasets were hierarchically clustered based on similarity in gene expression LogFC between HD and control samples. Height, or the distance metric between nodes, was calculated with the ward.D method. Abbreviations: CPu, caudate putamen; astro, astrocyte; epen, ependymal; endo, endothelial; IN, interneuron; PT, Pvalb/Th-expressing; iMSN/dMSN, indirect/direct pathway medium spiny neuron; MNS, MAP2+ and NES/SOX2-; OPC, oligodendrocyte progenitor cell; oligo, oligodendrocyte; micro, microglia; NSC, neural stem cell; Cg, cingulate cortex; FON, Foxp2/Olfm3-expressing neuron; SN, Sst/Npy-expressing; mono, monocyte; NPC, neural progenitor cell; CoNeuron, cortical neuron; Cd, caudate nucleus; pre, presymptomatic; GPC, glial progenitor cell; APC, astrocyte precursor cell; BBB, blood brain barrier.

**Figure S3. Overlap heatmap of exhaustive pairwise comparisons between 44 human HD DEG lists.** The number of overlapping DEGs is printed in the square, and the Fisher’s exact test p-value of the overlap is indicated by the color of the square. Note that the background gene set is not available for datasets Smith_hiPSC_MSN_NSC, Smith_hiPSC_MSN_MNS, Al_Cg_astro and Al_Cg_neuronal, and thereby no valid p values for comparisons involving these 4 datasets. Abbreviations: CPu, caudate putamen; astro, astrocyte; epen, ependymal; endo, endothelial; IN, interneuron; PT, Pvalb/Th-expressing; iMSN/dMSN, indirect/direct pathway medium spiny neuron; MNS, MAP2+ and NES/SOX2-; OPC, oligodendrocyte progenitor cell; oligo, oligodendrocyte; micro, microglia; NSC, neural stem cell; Cg, cingulate cortex; FON, Foxp2/Olfm3-expressing neuron; SN, Sst/Npy-expressing; mono, monocyte; NPC, neural progenitor cell; CoNeuron, cortical neuron; Cd, caudate nucleus; pre, presymptomatic; GPC, glial progenitor cell; APC, astrocyte precursor cell; BBB, blood brain barrier.

**Figure S4. LogFC heatmap of HD marker genes from Vashishtha et al. 2013 in 44 human HD datasets.** Each column is a HD dataset, each row is a HD marker gene. Both the HD datasets and the HD marker genes are clustered hierarchically. Abbreviations: CPu, caudate putamen; astro, astrocyte; epen, ependymal; endo, endothelial; IN, interneuron; PT, Pvalb/Th-expressing; iMSN/dMSN, indirect/direct pathway medium spiny neuron; MNS, MAP2+ and NES/SOX2-; OPC, oligodendrocyte progenitor cell; oligo, oligodendrocyte; micro, microglia; NSC, neural stem cell; Cg, cingulate cortex; FON, Foxp2/Olfm3-expressing neuron; SN, Sst/Npy-expressing; mono, monocyte; NPC, neural progenitor cell; CoNeuron, cortical neuron; Cd, caudate nucleus; pre, presymptomatic; GPC, glial progenitor cell; APC, astrocyte precursor cell; BBB, blood brain barrier.

**Figure S5. Enrichment heatmap of top 20 enriched gene sets of GO biological process or KEGG pathway in 44 human HD datasets by GSEA analysis.** Each column is a human HD dataset, each row is a gene set of GO biological process or KEGG pathway. Only gene sets with member number between 20 and 200 are kept, and are clustered hierarchically. Abbreviations: CPu, caudate putamen; astro, astrocyte; epen, ependymal; endo, endothelial; IN, interneuron; PT, Pvalb/Th-expressing; iMSN/dMSN, indirect/direct pathway medium spiny neuron; MNS, MAP2+ and NES/SOX2-; OPC, oligodendrocyte progenitor cell; oligo, oligodendrocyte; micro, microglia; NSC, neural stem cell; Cg, cingulate cortex; FON, Foxp2/Olfm3-expressing neuron; SN, Sst/Npy-expressing; mono, monocyte; NPC, neural progenitor cell; CoNeuron, cortical neuron; Cd, caudate nucleus; pre, presymptomatic; GPC, glial progenitor cell; APC, astrocyte precursor cell; BBB, blood brain barrier.

**Figure S6. LogFC heatmap of HD marker genes from Vashishtha et al. 2013 in 75 mouse and 4 other non-human datasets.** A&B. Each column or row is a dataset, each row or column is a HD marker gene. Both datasets and marker genes are clustered hierarchically based on LogFC with HD over WT. Abbreviations: CPu, caudate putamen; TRAP, translating ribosome affinity purification; iMSN/dMSN, indirect/direct pathway medium spiny neuron; CoNeuron, cortical neuron; astro, astrocyte; Ad-brown, brown adipose; Co, cortex; IN, interneuron; Ch, Cholinergic; FON, Foxp2/Olfm3-expressing neuron; CstrPN, corticostriatal projection neurons; micro, microglia; OPC, oligodendrocyte progenitor cell; epen, ependymal; oligo, oligodendrocyte; cc, corpus callosum; Ad-white-intest, instestinal white adipose; endo, endothelial; SN, Sst/Npy-expressing; PT, Pvalb/Th-expressing; HTH-TH, hypothalamus-thalamus; CA, hippocampus; Cb, cerebellum; Ad-white-gonad, gonadal white adipose.

**Supplementary Datasets**

**Dataset S1**. **Screening documentation of the HD systematic review.** This file contains the screening exclusion criteria, the PubMed article screening, the four data repository screening, the consolidation of data repository results, and the consolidation of PubMed and data repository results.

**Dataset S2. DEG lists of human HD data.** DEGs from the 50 human HD datasets were extracted using the FDR and LogFC cutoffs specified by the authors. For DEG lists representative of each human HD subcategory, 1) if there was only one dataset in that subcategory, that one DEG list was taken; 2) if there were two, the union of the two DEG lists were taken; 3) if there were more than two, DEGs that appeared in at least two of the datasets in that subcategory were taken. Category DEG lists were extracted based on gene frequency. Top 10 up-regulated and down-regulated DEG lists in each human dataset were extracted based on FDR.

**Dataset S3. DEG frequency in human HD subcategories/categories.** DEG membership is listed in 11 subcategories as well as cell culture and brain categories. HD dataset numbering is the same as in Dataset S2. Common and unique DEGs in a subcategory/category can be retrieved by filtering.

**Dataset S4. Intersection of two or more DEG lists within cell culture and brain categories.** Exhaustive pairwise and multiple comparisons between subcategories within cell culture and brain were made, with overlapping genes listed and Fisher’s exact test p values calculated.

**Dataset S5. GO biological process or KEGG pathway term enrichment analysis of common/unique DEG sets and regulator groups.** For multiple comparisons, the category background was used as the background; for pairwise comparisons, the overlap of backgrounds was used as the background; for unique DEGs, the background of the dataset where the unique DEGs were present was used as the background.

**Dataset S6. DEG list comparison between Lee_Cd_iMSN/Miller_blood_monocyte and all other primary tissues, SAT1 logFC in selected datasets, and concordance between RNA-seq and microarray data.** Overlapping and unique DEGs between iMSN/monocyte and other primary tissues were listed. SAT1 logFC values in selected datasets were listed. HD DEGs revealed by microarray and RNA-seq were compared.

**Dataset S7. Gene set enrichment analysis of 44 human HD DEG datasets.** Enrichment scores and false discovery rates were calculated for positively and negatively enriched gene sets of GO biological processes and KEGG pathways.

**Dataset S8. TF matrix of 44 human HD DEG datasets and concordance between TF score and H3K27ac ChIP-seq data.** TF score was calculated as the number of DEGs being a TF target divided by the total number of DEGs in that dataset. TFs were obtained from hTFtarget database and filtered by expression status from the closest cell/tissue type in the Human Protein Atlas. Transcription factor scoring analysis was performed on the HD iPSC Consortium 2017 study using the hTFtarget database. Concordance was checked between the TF lists and the H3K27ac ChIP-seq motif data from the same study. TFs were filtered by expression status in the Human Protein Atlas and colored by score levels.

**Dataset S9. Differentially expressed TFs and epigenetic modifiers in 44 human HD DEG datasets, expression status of predicted regulators and top 20 up-regulated and down-regulated regulators.** 1639 TFs from the Human TFs database and 167 epigenetic modifiers from the dbEM database were queried in the 44 human HD DEG datasets and their logFC values were retrieved if present. Regulators with differentially expressed targets were queried for their expression status in HD, and if differentially expressed the datasets where these regulators were up-regulated or down-regulated were counted. Among the differentially expressed predicted regulators, top 20 up-regulators and down-regulated were ranked based on 1) the number of datasets where the regulator is up-regulated minor down-regulated and 2) the sum of TF scores of the regulator in all datasets.

**Dataset S10.** **DEG analysis of mouse data, and comparison between mouse and human data.** DEG frequency/membership in early, intermediate and late HD stages is summarized in mouse datasets. DEG list comparison was made between early, intermediate and late HD stages. For the intermediate to late HD stages in mouse, striatal DEGs discovered by bulk RNA-seq were compared with MSN DEGs and interneuron DEGs discovered by snRNA-seq. Corresponding timepoints and cell types were compared between human and mouse: 1) early/presymptomatic striatal, 2) early/presymptomatic cortical, 3) intermediate-late/symptomatic MSN, 4) intermediate-late/symptomatic interneurons, and 5) intermediate-late/symptomatic cortical. Top 10 up-regulated and down-regulated DEG lists in each mouse dataset were extracted based on FDR.
